# Comparative Genomics of a Plant-Parasitic Nematode Endosymbiont Suggest a Role in Nutritional Symbiosis

**DOI:** 10.1093/gbe/evv176

**Published:** 2015-09-10

**Authors:** Amanda M.V. Brown, Dana K. Howe, Sulochana K. Wasala, Amy B. Peetz, Inga A. Zasada, Dee R. Denver

**Affiliations:** ^1^Department of Integrative Biology, Oregon State University; ^2^USDA-ARS Horticultural Crops Research Laboratory, Corvallis, Oregon

**Keywords:** comparative genomics, nutritional symbiosis, Verrucomicrobia, *Wolbachia*, *Akkermansia*, *Xiphinematobacter*

## Abstract

Bacterial mutualists can modulate the biochemical capacity of animals. Highly coevolved nutritional mutualists do this by synthesizing nutrients missing from the host’s diet. Genomics tools have advanced the study of these partnerships. Here we examined the endosymbiont *Xiphinematobacter* (phylum Verrucomicrobia) from the dagger nematode *Xiphinema americanum*, a migratory ectoparasite of numerous crops that also vectors nepovirus. Previously, this endosymbiont was identified in the gut, ovaries, and eggs, but its role was unknown. We explored the potential role of this symbiont using fluorescence in situ hybridization, genome sequencing, and comparative functional genomics. We report the first genome of an intracellular Verrucomicrobium and the first exclusively intracellular non-*Wolbachia* nematode symbiont. Results revealed that *Xiphinematobacter* had a small 0.916-Mb genome with only 817 predicted proteins, resembling genomes of other mutualist endosymbionts. Compared with free-living relatives, conserved proteins were shorter on average, and there was large-scale loss of regulatory pathways. Despite massive gene loss, more genes were retained for biosynthesis of amino acids predicted to be essential to the host. Gene ontology enrichment tests showed enrichment for biosynthesis of arginine, histidine, and aromatic amino acids, as well as thiamine and coenzyme A, diverging from the profiles of relatives *Akkermansia muciniphilia* (in the human colon), *Methylacidiphilum infernorum*, and the mutualist *Wolbachia* from filarial nematodes. Together, these features and the location in the gut suggest that *Xiphinematobacter* functions as a nutritional mutualist, supplementing essential nutrients that are depleted in the nematode diet. This pattern points to evolutionary convergence with endosymbionts found in sap-feeding insects.

## Introduction

Microbial mutualists are increasingly understood to be widespread in animal species, probably because they expand the biochemical repertoire of the hosts (Douglas 2009; Gerardo and Wilson 2011; Ferrari and Vavre 2011; Hussa and Goodrich-Blair 2013). Thus, their biological importance and biochemical integration can be significant in the adaptation of organisms and a major force in the evolution of biological systems (Dethlefsen et al. 2007; Ellers et al. 2012; Wernegreen 2012; McFall-Ngai et al. 2013). For example, mitochondria and plastids contributed major innovations in early eukaryotes, and highly coevolved obligate symbioses shift the flow of carbon, nitrogen, and organics, by providing photosynthesis to nonphotosynthetic organisms (e.g., in lichens and corals) or by assimilating elements (e.g., nitrogen-fixing bacteria in legumes) or breaking down organic compounds (e.g., cellulose digesting microbes in herbivore guts). Bacteria-animal endosymbioses have arisen at least a dozen times from at least five bacterial phyla, often serving in biosynthesis of nutrients missing in the host diet (Toft and Andersson 2010; Moran and Bennett 2014). These endosymbioses are common in the sap- and blood-feeding insects, but may occur more broadly among invertebrates that are pests of plants or parasites with specialized diets (Gottlieb et al. 2008, 2012; Douglas 2009; Bright and Bulgheresi 2010). So far, it has been challenging to identify and characterize these associations due to difficulty culturing and manipulating the microbes; however, genomic tools now offer a promising approach for simultaneous discovery and functional analysis of vital microbial partners.

Nematodes have diverse ecological roles and recently have been shown to harbor varying communities of microbial symbionts (De Ley 2006; Murfin et al. 2012; Baquiran et al. 2013; Cheng et al. 2013). Plant-parasitic nematodes cause about $100 billion in global annual agricultural damage, and also contribute to terrestrial carbon flow through the rhizosphere (Neher 2001, 2010; Piśkiewicz et al. 2007; Jones et al. 2013). Plant-parasitic nematodes have evolved from free-living nematodes at least three times (Van Megen et al. 2009; Haegeman et al. 2011). During these transitions from an ancestral diet generally feeding on bacteria to a plant-based diet, some nematodes met the challenge of the new food source through horizontal gene transfers, for example, cellulases in the Tylenchida (Danchin et al. 2010; Haegeman et al. 2011; Paganini et al. 2012). In the Tylenchida, as well as in other invertebrates, these horizontally transferred genes are thought to have arisen from past symbioses that have since been lost (Hotopp et al. 2007; Husnik et al. 2013; Koutsovoulos et al. 2014). In nematodes, mutualistic endosymbiotic associations with *Wolbachia* appear to have played a role in the transition to a new diet for filarial nematodes in mammal hosts, for example, *Brugia malayi*, the cause of lymphatic filariasis, and possibly historically for strongyloidean parasites in cattle, for example, *Dictyocaulus viviparus* (McNulty et al. 2010; Comandatore et al. 2013; Koutsovoulos et al. 2014; Zug and Hammerstein 2014).

Here we examine an endosymbiont of the dagger nematode, *Xiphinema americanum* species complex (hereafter, *X. americanum*), so named for its long protruding spear-like mouthpart, or odontostyle used to penetrate host-plant roots. *Xiphinema americanum* is a large species-complex comprising about 36 named species forming several major clades (Lamberti and Ciancio 1993; Zasada et al. 2014). These species are migratory ectoparasites of plant roots and serious pests of hundreds of plants, including corn, soybeans, fruits, grapevines, tomato, tobacco, and coffee (http://www.ipm.ucdavis.edu/NEMABASE/, last accessed January 1, 2014). Much of their damage is caused by transmission of nepoviruses (single-stranded RNA plant-viruses) (Taylor and Brown 1997). *Xiphinema americanum* harbor intracellular bacterial endosymbionts with an unknown role: *Candidatus Xiphinematobacter americani*, *Candidatus Xiphinematobacter rivesi*, and *Candidatus Xiphinematobacter brevicolli* (Vandekerckhove et al. 2000); hereafter referred to collectively as *Xiphinematobacter.* Previous authors have suggested that *Xiphinematobacter* may cause parthenogenesis (Coomans and Claeys 1998; Coomans and Willems 1998; Luc et al. 1998), based on exclusive association with species in the *X. americanum* species-complex which almost always use thelytokous parthenogenesis rather than sexual reproduction, but they also occur in the gut where they could play a nutritional role (Coomans et al. 2000; Vandekerckhove et al. 2000, 2002).

Our comparative genomics study centers around *Xiphinematobacter*, which belongs to the Verrucomicrobia, a recently described bacterial phylum whose closest relatives are the Planctomycetes and Chlamydiae (Schlesner et al. 2006). These species have wide-ranging morphologies and metabolisms (Sangwan et al. 2004; Schlesner et al. 2006; Hou et al. 2008; Van Passel et al. 2011; Wertz et al. 2012; Herlemann et al. 2013). This group was reported to be abundant, making up 11–23% of soil bacteria (Bergmann et al. 2011). Members with host associations are quite diverse. Although *Xiphinematobacter* is obligately intracellular (Coomans and Claeys 1998; Coomans and Willems 1998; Coomans et al. 2000; Vandekerckhove et al. 2000), other Verrucomicrobia range from free-living, to extracellular in the host lumen (e.g., *Akkermansia muciniphilia*—a mucin-degrader in the human colon inversely associated with obesity; Derrien et al. 2004; Van Passel et al. 2011), to intranuclear (e.g., *Candidatus Nucleococcus trichonymphae*—in the nuclei of protists in termite guts; Sato et al. 2013). This diversity of host-associations and the availability of several complete genomes from Verrucomicrobia allowed us to compare genetic patterns among free-living and host-associated relatives to explore the potential role of the endosymbiont *Xiphinematobacter* in *X. americanum*.

Our study presents fluorescence in situ hybridization (FISH) microscopy analysis coupled with the complete genome of the endosymbiont *Xiphinematobacter* from the nematode *X. americanum*, along with a comparative functional genomics analysis to illuminate the symbiont’s role. Our findings are novel in that this is the first report of the complete genome sequence from a non-*Wolbachia* exclusively intracellular endosymbiont in a nematode and the first genome from an intracellular Verrucomicrobium. Together, our results support a nutritional mutualist role for this endosymbiont much like that found in distantly related endosymbionts from sap-feeding insects. Such findings could have enormous implications for understanding the role of this symbiont in host-plant specificity of these nematodes (Hansen and Moran 2014).

## Materials and Methods

### Nematode Collection

Nematodes identified morphologically as *X. americanum* originally collected from grape (*Vitis vinifera*) in Idaho, USA were cultured in the greenhouse on sorghum-sudangrass (*Sorghum sudanense*) at the USDA-ARS in Corvallis, OR. Nematodes were extracted from culture soil using a combination of decant-sieving and sugar centrifugation (Ingham 1994). Approximately 1,000 individuals comprising mostly adult females and juveniles were collected from the extracted soil samples for genomic library preparations and another 30 individuals of mixed stages, and also individual eggs containing embryos of various stages, were collected for FISH microscopic study. Because these long-lived nematodes cannot be easily grown as single-maternal lineages, our pooling strategy of combining many nematodes for sequencing can include multiple genotypes or strains.

### FISH and Confocal Microscopy

FISH was performed largely following the high-stringency protocol of Vandekerckhove et al. (2002). Mixed stages, including juveniles, females, and eggs were surface sterilized in 100 µl 0.1% wt/vol benzalkonium chloride for 1 min, then washed twice with 100 µl of 0.85% wt/vol NaCl for 2 min and permeabilized by fixation in 100 µl of 1:1 vol/vol glacial acetic acid and 100% ethanol for 10 min, washing twice in 100 µl of 100% ethanol for 5 min. Samples were then treated with a 10-min soak in 100 µl of 1:1 vol/vol 100% methanol and phosphate-buffered saline solution with Tween (PBT; 150 mM NaCl, 10 mM Na_2_HPO_4_, 0.1% wt/vol Tween 20, with HCl to adjust to pH 7.4). Specimens were then fixed in 100 µl of 1% vol/vol formaldehyde and PBT for 30 min, followed by two washes in 100 µl of PBT for 2 min. Hybridization was performed for 3 h at 46 °C using 90 µl of prewarmed hybridization buffer (20 mM Tris–HCl pH 7.4, 0.02% wt/vol sodium dodecyl sulfate [SDS], 0.9 M NaCl, 5 mM ethylenediaminetetraacetic acid [EDTA], 60% vol/vol formamide) and 10 µl of the probe at 10 µM in TE (10 mM Tris–HCl, 1 mM EDTA, pH 7.4). The FISH probe 5′-/5ATTO633N/TGC TGC CAC CCG TAG GTG T-3′ was designed to target the 16S rRNA of Verrucomicrobia and was based on the probe EUB338-III (Daims et al. 1999), but modified to accommodate an ATTO fluorophore by adding a T to the 5′-end. After hybridization, specimens were washed twice at 48 °C for 30 min in 100 µl of prewarmed hybridization wash buffer (20 mM Tris–HCl, 0.02% wt/vol SDS, 0.008 M NaCl, 5 mM EDTA). These conditions were previously shown to ensure high-specificity (Vandekerckhove et al. 2002). Finally, specimens were mounted on slides in 8 µl of VECTASHIELD with DAPI (Vector Laboratories, Burlingame, CA) and viewed on a Zeiss LSM510 META with Axiovert 200 motorized microscope with version 3.2 LSM software at the Center for Genome Research and Bioinformatics (CGRB; Oregon State University, Corvallis, OR). Negative controls for FISH were prepared as above using 1) the reverse compliment of the probe, 2) no probe during hybridization, and 3) *Caenorhabditis briggsae* instead of *Xiphinema americanum*.

### DNA Library Preparation and Sequencing

DNA from 1,000 *X. americanum* was prepared using Qiagen DNeasy Blood & Tissue Kit (Valencia, CA) following an initial grinding step using a motorized micropestle for 2 min to disrupt the cuticles. Genomic library preparation was performed using the Illumina TruSeq DNA Sample Preparation Kit (San Diego, CA) following the manufacturer’s instructions, with initial shearing using a Diagenode Bioruptor Pico (Denville, NJ) for 50 s, to obtain peak library fragment sizes of approximately 600–700 bp. Following adapter ligation, fragmented ligated targets about 650–750 bp were gel-excised. Paired-end sequencing was performed using the Illumina MiSeq system for 301 cycles at the CGRB.

### *Xiphinematobacter* Genome Assembly and Polymerase Chain Reaction Finishing

Raw reads were inspected for quality drop off, and then trimmed accordingly using a custom perl script. Reads were then quality filtered using FASTX-Toolkit v.0.014 (http://hannonlab.csht.edu/fastx_toolkit/) and the genome assembly was performed using Velvet v.1.2.10 (Zerbino and Birney 2008) with parameters such as kmer size, coverage cutoff and average coverage optimized to select for the endosymbiont by looking for the largest contig sizes and greatest total contig lengths matching genomes from phylum Verrucomicrobia by BLAST+ v.2.2.29 (NCBI; National Center for Biotechnology Information) searches. This produced two large scaffolds 930,828 and 64,662 bp with an approximately 100-bp overlap, with numerous stretches of Ns, and several shorter scaffolds with lower read-depth coverage that were 99% similar to regions within the larger scaffold. GapFiller v.1-11 (Boetzer and Pirovano 2012) was used to help close gaps with “Ns.” The resulting linear scaffolds were further inspected using bwa and SAMtools (Li et al. 2009; Li and Durbin 2009) mapping all reads to the assembly and inspecting the .bam file using the tablet v.1.12.03.26 genome viewer (Milne et al. 2010) to look for regions of uneven coverage, Ns, or unpaired reads suggestive of assembly errors. Because the 16S rRNA gene was found in two separate contigs at 99% similarity, BLAST (Basic Local Alignment Search Tool) searches were performed for several other genes and this showed the same pattern, suggesting the presence of two strains of *Xiphinematobacter*. Thus, systematic assembly errors (duplicated regions due to a second low-coverage strain) were removed manually as follows. Mapping was performed to show distinctly different coverage depth between duplicates on either side of Ns, suggestive of artifacts resulting from two strains (supplementary fig. S1, Supplementary Material online). To assemble the high-coverage strain, the lower-coverage duplicates were removed, GapFiller was run again, then finally reads were mapped back to the contigs and the final assembly was inspected in tablet to check for coverage discrepancies, duplicates, Ns, and unpaired reads. Final genome-finishing to close the circular genome and confirm the overlap of the two scaffolds was performed using polymerase chain reaction (PCR) primers (supplementary table S1, Supplementary Material online) and sequencing was done using BigDye Terminator v. 3.1 Cycle Sequencing Kit (Applied Biosystems) using an ABI Prism 3730 Genetic Analyzer at the CGRB. Although the focus in this study was the more abundant strain, we also assembled and analyzed the less abundant strain as follows. De novo genome assembly was performed optimizing for lower coverage in velvet. The resulting Verrucomicrobial contigs were ordered in Mauve 2.3.1 multiple sequence aligner (Darling et al. 2010) using the abundant strain as a reference to enable an analysis of gene order differences. Because of the difficulty obtaining a complete, contiguous de novo assembly for the second strain, strain divergence was analyzed by a mapping approach. Filtered reads were mapped to the finished genome of the common strain using bwa and SAMtools, then single nucleotide polymorphisms (SNPs) and indels were called using the Genome Analysis Toolkit GATK v.3.3-0 (DePristo et al. 2011) pipeline. Gene content from the strains was compared by generating a variant genotype from the .vcf using vcftools (Danecek et al. 2011) and aligning this to the reference. Distribution of SNPs with respect to coding positions was calculated using KaKs-calculator v.1.2 (Zhang et al. 2006). To determine whether both strains were present in single nematodes, single-nematode DNA preparations were performed as described in Zasada et al. (2014), followed by PCR (see supplementary table S1, Supplementary Material online) and BigDye sequencing (described above).

### Gene Annotation and Functional Category Assignment

The Prokka package v1.10 (Seemann 2014) was used to perform complete genome annotation for *Xiphinematobacter* and the other bacteria listed in table 2. Briefly, Prokka employs BioPerl, Prodigal for ab initio gene prediction, HMMER3 for protein family profiles, BLAST+ for comparative annotation, RNAmmer/Barrnap for rRNAs, Aragorn for tRNAs, Infernal for ncRNAs, and SignalP for signal peptide prediction. For outgroup bacteria, gene lists resulting from these annotations were inspected against NCBI GenBank annotations using Artemis (Carver et al. 2012) and any genes that were missed by genome annotation or duplicated by Prokka were corrected. Resulting gene lists were further inspected and sorted into functional categories (COG: clusters of orthologous groups; and GO: gene ontology) using GoMiner v291 (Zeeberg et al. 2003) with the taxon IDs enriched for Verrucomicrobia (361055, 2736, 239935, and 191863) or *Wolbachia* (77551, 100901, 80849, and 63437), and using the KEGG database (www.genome.jp/kegg/pathway.html) for pathway analysis/mapping. Annotation was repeated using the same version of Prokka for all other species listed in table 2. These outgroup species consisted of 13 Verrucomicrobia species with complete or relatively good quality draft genomes available in GenBank, representing the widest possible range of phylogenetic and lifestyle (habitat) states, based on previous studies. Two bacterial species were also chosen from sister clades to the Verrucomicrobia as outgroups, Planctomycetes and Chlamydiae. For comparative genomics of gene reduction and mutualism, we also included two strains of *Wolbachia* representing 1) a large genome-size reproductive manipulator from the arthropod-host supergroups (A/B), wPip, and 2) a small genome-size mutualist from the filarial nematode-host supergroups (C/D), wOo. Metabolic pathways were predicted and reconstructed based on conserved pathways in the KEGG and MetaCyc (Caspi et al. 2014) databases and UniProtKB (UniProt Consortium 2014) together with pathways predicted for endosymbionts (Shigenobu et al. 2000; Koga and Moran 2014; Nikoh et al. 2014; Santos-Garcia, Latorre et al. 2014).

**Table 2:** Habitat and Genomic Features of Endosymbiont *Xiphinematobacter* Compared with Other Verrucomicrobia and Outgroup Bacteria Available in GenBank

Species	Accession^a^	Habitat	Genome Size	Proportion Coding	%GC	Proteins	rRNA Operons	tRNAs	Ortholog Length
*Xiphinematobacter americani*	CP012665	Endosymbiotic, in plant-parasitic nematodes	915,884	0.84	47.7	817	1	45	25,047
*Akkermansia muciniphilia*	NC_010655.1	Human colon, beneficial	2,664,102	0.89	55.8	2,182	3	54	25,578
*Rubritalea marina*	NZ_ARJP00000000.1	Marine, associated with sponge	3,013,001	0.91	51.5	2,655	2	43	25,321
*Diplosphaera colitermitum*	ABEA00000000.3	Termite gut, culturable	5,671,497	0.87	60.9	5,023	1	68	25,433
*Verrucomicrobiae bacterium *DG	NZ_ABSI00000000.1	Marine, associated with dinoflagellate	5,769,153	0.90	54.3	4,684	1	45	25,231
*Methylacidiphilum infernorum*	NC_010794.1	Free-living, extreme acidophile/thermophile	2,287,145	0.89	45.5	2,101	1	46	25,272
*Verrucomicrobium* sp. 3C	NZ_ARAS00000000.1	Free-living, lakes	2,772,066	0.87	60.9	2,727	1	48	25,304
*Coraliomargarita akajmensis*	NC_014008.1	Free-living, near corals	3,750,771	0.90	53.6	3,138	2	47	25,198
*Verrucomicrobia bacterium* SC	NZ_ARTV00000000.1	Free-living, marine	3,949,105	0.87	47.7	3,272	2	28	25,521
*Opitutus terrae*	NC_010571.1	Free-living, rice paddy soil	5,957,605	0.89	65.6	4,669	1	72	25,410
*Opitutaceae bacterium*	NZ_AHKS00000000.2	Free-living, termite associated	7,067,882	0.88	62.1	5,899	1	71	25,765
*Pedosphaera parvula*	NZ_ABOX00000000.2	Free-living, pasture soil	7,414,222	0.89	52.6	6,411	2	60	25,414
*Chthoniobacter flavus*	NZ_ABVL00000000.1	Free-living, soil	7,848,700	0.88	61.1	6,667	2	64	25,383
*Verrucomicrobia spinosum*	NZ_ABIZ01000001.1	Free-living, aquatic/soil/Feces	8,220,857	0.87	60.3	6,502	4	67	25,646
*Parachlamydia acanthamoebae*	NC_015702.1	Lives in acanthamoebae, human pneumonia	3,072,383	0.89	39.0	2,569	5	40	25,359
*Lentisphaera araneosa*	NZ_ABCK00000000.1	Free-living, marine	6,023,180	0.88	40.9	4,903	6	42	25,431
*Wolbachia* sp. wPip	NC_010981.1	Endosymbiotic, reproductive parasite of insects	1,482,455	0.88	34.2	1,423	1	34	NA
*Wolbachia* sp. wOo	NC_018267.1	Endosymbiotic, mutualist of filarial nematodes	957,990	0.70	32.1	881	1	32	NA

Note.—Species details: Verrucomicrobial strains are *A. muciniphilia* ATCC BAA-835, *R. marina* DSM 17716, *D. colitermitum* TAV2, *V. bacterium* DG1235 ASM15569v1, *M. infernorum* V4, *V. bacterium* SCGC AAA164-E04 ASM38371v1, *O. terrae* PB90-1, *O. bacterium* TAV1 ASM24349v3, *P. parvula* Ellin514, *Ch. flavus* Ellin428, *V. spinosum* DSM 4136 = JCM 18804. Outgroup strains are Phylum Chlamydiae *P. acanthamoebae* UV-7, Phylum Lentisphaerae *L. araneosa* HTCC2155, *Wolbachia* sp. wPip endosymbiont of *Culex quinquefasciatus* Pel, *Wolbachia* sp. wOo endosymbiont of *Onchocerca ochengi* ASM30688v1.

^a^RefSeq Accession or International Nucleotide Sequence Database Collaboration (WGS).

### Comparative Synteny Analysis

Initial comparisons of gene order conservation between *Xiphinematobacter* and other Verrucomicrobia were made using the Mauve. Because of large sequence divergence and low gene order conservation in this phylum, further analyses were performed to identify and characterize regions with conserved gene order between *Xiphinematobacter* and outgroups by first finding all regions with proteins that align among outgroups *Chthoniobacter flavus*, *Opitutus terrae*, and *Pedosphaera parvula* using promer from MUMmer 3.1 (Kurtz et al. 2004), and identifying all blocks of two or more genes with shared order among these species. These conserved synteny blocks were compared against the annotated *Xiphinematobacter* genome.

### Orthologous Gene Comparisons and Phylogenomic Analysis

From the 16 verrucomicrobial species listed in table 2, a set of single-copy orthologous genes were chosen based on BLAST matches and PROKKA and these were translated and aligned in Geneious v.5.4.4 (created by Biomatters). Thereafter, maximum-likelihood trees were reconstructed using RAxML-HPC2 v.8.0.24 (Stamatakis 2006) run on XSEDE (CIPRES Science Gateway V 3.1 http://www.phylo.org/sub_sections/portal/) using the PROTCATDAYHOFF substitution model and empirical amino acid frequencies with likelihood evaluation under the GAMMA model and free model parameters estimated by RAxML with fast-bootstrapping (1,000 replicates).

### Gene Enrichment Tests and Comparative Genomics

Gene set enrichment comparisons were performed to examine patterns that may be associated with differential gene loss or retention due to the environment, thus we chose three target verrucomicrobial species: *Xiphinematobacter*, *A. muciniphilia*, and *Methylacidiphilum infernorum*. These species originated from three distinct environments, the first two species being host-associated in very different hosts (plant-parasitic nematode vs. human intestine), whereas the third is free-living in extreme thermophilic/acidophilic environments. These species also have the smallest genomes (table 2). We used an outgroup gene set comprised a set of overlapping, nonredundant genes shared among three free-living Verrucomicrobia, which occur on different branches of the tree: *Ch. flavus*, *O. terrae*, and *Pedosphaera parvula*. This shared gene set from widely diverged free-living Verrucomicrobia was used in enrichment tests against each target gene set. Gene enrichment tests for *Wolbachia* involved a comparison of two strains: The target gene set from the obligate mutualist *Wolbachia* strain wOo from a filarial nematode and from the reproductive manipulator *Wolbachia* strain wPip from a mosquito. Functional enrichment tests were performed using the one-sided Fisher exact *P* value, testing the proportion of genes in a GO category for the target species (e.g., *Xiphinematobacter*) relative to the expected proportion of genes in that category, comprised the total gene set from the target species and the gene set from the shared outgroups. Fisher exact *P* values were generated using the GoMiner software (Zeeberg et al. 2003) with multiple testing correction. GoMiner correction for multiple testing (false discovery rate or FDR) resamples the *P*-value distribution based on the number of genes in a category, and is quite overconservative (high rate of type II error) for categories with small numbers of genes (Zeeberg et al. 2003; and see supplementary material, Supplementary Material online ). It also overestimates GO categories (i.e., it annotates many functions even for pathways, processes, or cellular components that do not exist in the test organism); thus, FDR is also somewhat overconservative globally. For these reasons, we report both FDR-corrected and uncorrected *P* values.

## Results

### *Xiphinematobacter* Localization by FISH

**Fig. 1.—:**
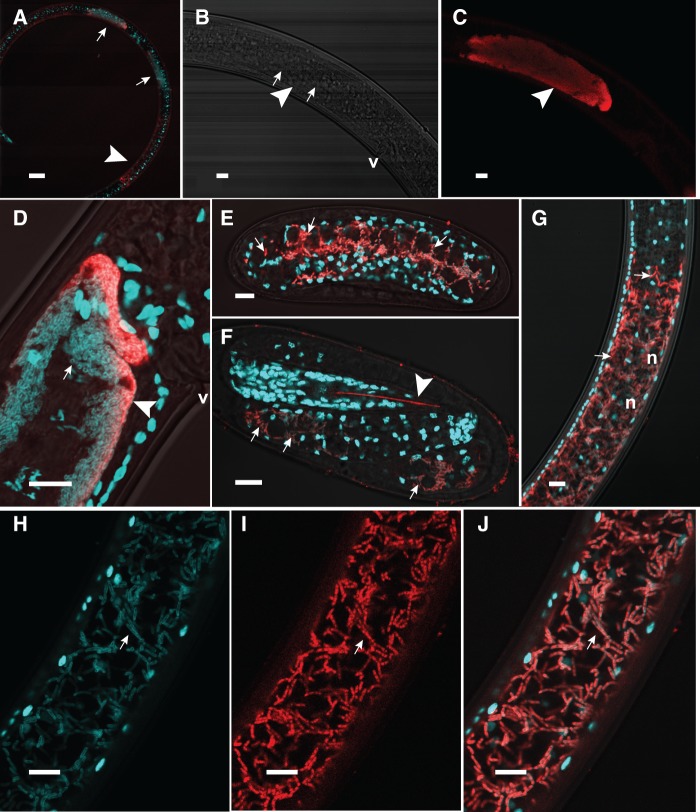
FISH images of the endosymbiont *Xiphinematobacter* within adult female nematode *Xiphinema americanum* ovaries, developing eggs, and gut lining. Whole mount of adult female nematode (*A*) with the endosymbionts (red) within paired ovaries (small arrows) and gut (large arrowhead). Ovaries (*B–D*) with ovarian sheath (large arrowheads) filled with endosymbionts stained by both DAPI (blue) and FISH-probe (red) (v, nematode vulva). Images (*B*) and (*C*) depict the same confocal image with DAPI-only and FISH-probe-only wavelengths, respectively. Enlarged nuclei of developing eggs in (*B*) (small arrows) are not visible in probe-only image (*C*). Developing early (*E*) and late (*F*) embryos filled with diffuse and localized endosymbionts in the gut primordia (small arrows in [*E*] and [*F*], respectively). Developing odontostyle in (*F*) autofluorescences in red but not blue range (large arrowhead). Gut region of nematode (*G–J*) with elongate and dividing endosymbionts within host cells (small arrows) of the gut wall (n, nematode nuclei surrounded by endosymbionts with weaker fluorescence in the far confocal plane). Endosymbionts within gut wall cells for same confocal image with DAPI-only (*H*), FISH-probe-only (*I*), and DAPI-FISH-brightfield overlaid (*J*). Scale bar (*A*) = 50 µm. Scale bars (*B–J*) = 10 µm.

FISH results (fig. 1*A*–*J*) showed that the FISH probe was localized specifically to bacterial cells within the nematode gut wall, ovarian sheath, eggs, and developing embryos. The DAPI stain colocalized with the probe consistently, highlighting bacterial cells of two forms corresponding to their location: Rod-shaped, larger cells, about 4–7 µm long × 1.0 µm diameter, within cells of the gut (fig. 1*G*–*J*), and smaller, densely packed rod-shaped to coccoid cells, about 1.9–2.1 µm long × 0.7–0.9 µm diameter within the ovaries (fig. 1*B*–*D*). The longer appearance of bacterial cells in the gut may be partially due to cell division with daughter cells often not fully separated by cytoplasmic constriction or by new cells remaining close together. Three-dimensional rotation using the Zeiss confocal microscope z-stack feature allowed us to confirm the absence of these cells in the lumen of the gut. In eggs containing early and late embryos (fig. 1*E* and *F*), the probe localized to small rod-shaped or coccoid cells about 4 µm long × 0.7 µm in diameter. In later embryos with more defined first-stage juvenile features, the probe localized to focal areas consistent with regions that may develop into the gut. Negative controls (probe with *C. briggsae*, and reverse-complemented probe, or no probe with *X. americanum*) produced only very weak, diffuse fluorescence localized to the cuticle and strong autofluorescence of the odontostyle and sometimes amorphous and uneven-shaped specs outside the nematodes, similar to that seen in figure 1*F*.

### Full Genome of Endosymbiont *Xiphinematobacter* Sequenced at High Coverage

We generated a complete, circular genome for the endosymbiont *Xiphinematobacter*. The Illumina MiSeq results and assembly details are shown in table 1. Initial assemblies produced closest BLAST matches for bacterial 16S rRNA to *Candidatus Xiphinematobacter rivesi* (98% similarity), *Candidatus Xiphinematobacter americani* (96%), and *Candidatus Xiphinematobacter brevicolli* (94%). The finished endosymbiont genome was 915,884 bp long and sequenced at high coverage (approximately 1,290× read depth). It revealed 47.7% GC content, 817 predicted proteins (147 of these annotated as “hypothetical protein”), 45 tRNAs, and one copy of each typical rRNA gene. There were 4 predicted pseudogenes and 33 predicted signal peptides (supplementary table S2, Supplementary Material online). There was little repetitive DNA, except for two tandem repeat regions (9 repeats of 75 bp at position 60,350; 12 repeats of 15 bp at position 914,875), both causing assembly breaks that had to be closed by PCR.

**Table 1:** Sequencing and Assembly Data for the Endosymbiont *Xiphinematobacter* in the Plant-Parasitic Nematode *Xiphinema americanum*

Number of Illumina Reads	Number of Mapped Reads	Scaffold N50	Number of Scaffolds	Final Genome Size (bp)	Average Coverage Depth
15,133,970	4,316,327	921,693^a^	2	915,884	1,373

^a^This *Xiphinematobacter* scaffold was larger than the final genome because initial assembly included duplicated regions representing a second strain.

The data revealed two slightly diverged strains of *Xiphinematobacter* at 99.4% sequence similarity, with the abundant strain at approximately 1,050× and the less abundant strain at approximately 240×. De novo assemblies of the strains did not reveal any difference in gene order (supplementary fig. S3, Supplementary Material online). Between strains there were 5,676 SNP differences and one 5-bp deletion, with no change in gene content and no predicted additional pseudogenes. Distribution of SNPs was relatively even across the genome (supplementary table S4 and fig. S2, Supplementary Material online). The rate of nonsynonymous substitutions compared with synonymous substitutions (Ka/Ks) between strains was generally less than 1 (supplementary fig. S3, Supplementary Material online), with a mean of 0.27 across all genes. Genes for essential amino acid biosynthesis pathways had a mean Ka/Ks of 0.21 (see supplementary fig. S3, Supplementary Material online). Sequencing of a variable region from 18 individual nematodes (see supplementary table S1, Supplementary Material online), including 6 SNPs that distinguish the strains, revealed 15 individuals matching the abundant strain and 3 individuals matching the less abundant strain, with no signs of secondary peaks in the sequence trances at the variant positions.

### Verrucomicrobia and Outgroups Show Correlations in Genome Streamlining

Following genome annotation of the predominant *Xiphinematobacter* strain in the sample (deposited in NCBI GenBank under accession number CP012665), genomes were compared and show that the endosymbiont *Xiphinematobacter* has the smallest genome of any Verrucomicrobia, with the fewest proteins (table 2). Among Verrucomicrobia and outgroups listed in table 2, there was a positive relationship between number of proteins and genome size, and the length of noncoding sequence and genome size (supplementary fig. S4, Supplementary Material online), with the endosymbiont *Xiphinematobacter* having the most reduced genome size, protein number, and noncoding length, even compared with the endosymbiotic nematode mutualist *Wolbachia* wOo. Results also showed slight positive relationships with genome size for number of tRNAs, average length of proteins, and lengths of 81 conserved orthologous genes (the set used in phylogenomic tree reconstruction) (supplementary fig. S4, Supplementary Material online), with *Xiphinematobacter* displaying the shortest orthologous gene length. In contrast, average intergenic space did not show a positive relationship with genome size (supplementary fig. S4, Supplementary Material online) and *Xiphinematobacter* and *Wolbachia* wOo had larger predicted intergenic spaces than other species. There was no obvious clustering among host-associated or free-living Verrucomicrobia for any genomic feature.

### Phylogenomic Tree Supports Mixed Pattern of Host-Association

**Fig. 2.—:**
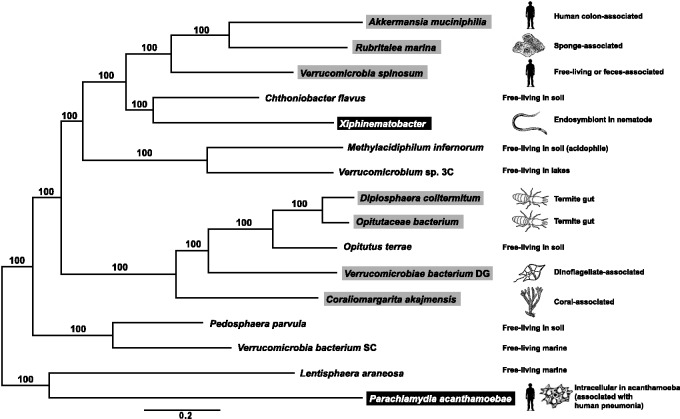
Maximum-likelihood tree generated with RAxML showing the relationship of the endosymbiont *Xiphinematobacter *to other verrucomicrobial species and two outgroup species based on protein sequences from 81 orthologous genes, comprising 29,967 alignment positions. Percent bootstrap values from 1,000 replicates are indicated on branches. Host-associations are indicated with icons on the right (human, sponge, nematode, termite, dinoflagellate, coral, acanthamoebae), and type of host-association is indicated by boxes around species names (black, endosymbionts; gray, extracellular host-associations). Others are free-living with habitat indicated as text on right.

From all annotated genes for the 14 Verrucomicrobia and 2 outgroup clades shown in table 2, 81 predicted single-copy orthologs were found (see list in supplementary table S5, Supplementary Material online). Nucleotide sequence alignment was difficult except between close relatives, so phylogeny reconstruction was performed using translated amino acid sequences that were predicted to have fewer saturated sites. The alignment is shown in supplementary table S6, Supplementary Material online. The resulting maximum-likelihood phylogeny (fig. 2) was well supported by bootstrap at all nodes. Intracellular host-associated taxa had slightly longer branches than their most closely related free-living taxa. Free-living and host-associated species occurred in a mixed pattern, suggesting independent origins of host-association. Verrucomicrobia formed several clades consistent with previous phylogenetic studies comprising more species but fewer loci (Sangwan et al. 2004; Schlesner et al. 2006). Clades include one basal with soil/marine free-living species, one with both termite-associated and marine-host associated species (subdivision 4 of Hugenholtz et al. 1998), a third clade with the nematode endosymbiont *Xiphinematobacter* and the sister-species *Ch. flavus* (subdivision 2, or “Spartobacteria” of Sangwan et al. 2004), and a sister clade with human- and sponge-associated species (subdivision 1, or “Verrucomicrobiae” of Sangwan et al. 2004).

### Genome Content and Architecture

**Fig. 3.—:**
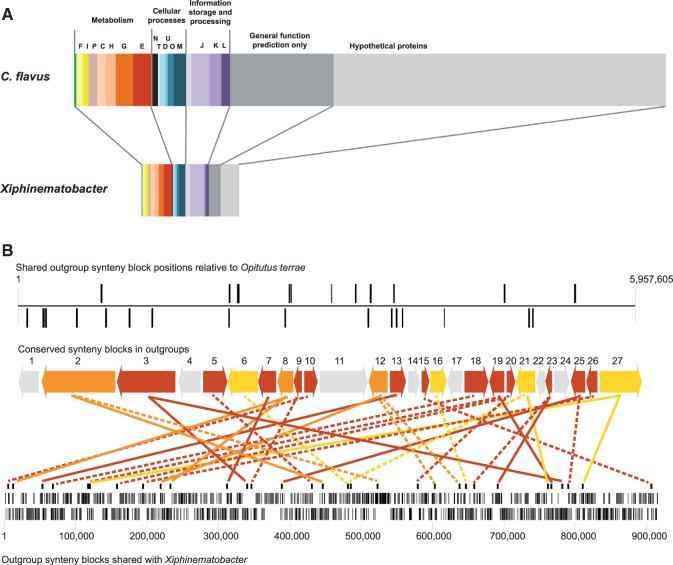
General genome architecture of *Xiphinematobacter* compared with outgroups. (*A*) Comparison of the number of genes in the endosymbiont *Xiphinematobacter* with its free-living relative, *Ch. flavus*, sorted by functional category (COG). (*B*) Conserved gene order (synteny) between *Xiphinematobacter* and other Verrucomicrobia. Top: All synteny blocks having two or more genes in the same order in *Ch. flavus*, *O. terrae*, and *Pedosphaera parvula* (shown relative to *O. terrae*) in forward or reverse orientation (vertical bars above and below line, respectively); center block-arrows depict relative sizes and orientations of 27 conserved synteny blocks shown at top, with colors depicting genes retained in *Xiphinematobacter* synteny blocks: Gray, no genes retained; red, all genes retained; orange, one gene missing; yellow, two genes missing. Solid and dashed lines beneath block-arrows connect to the same synteny blocks in the *Xiphinematobacter* genome shown at bottom (solid and dashed lines: forward and reversed orientations, respectively). Bottom: *Xiphinematobacter* genome with vertical bars indicating coding regions on the forward (top) and reverse (bottom) strands. (See complete list of genes in synteny blocks in supplementary table S3, Supplementary Material online.).

*Xiphinematobacter* had a greater proportion of genes well-annotated categories compared with its closest relative with an available genome, *Ch. flavus*, which had a larger proportion of genes with no assignment (no homologs, listed “hypothetical protein” or “general function prediction only”) (see fig. 3*A*). The COG categories that were most increased in proportion in *Xiphinematobacter* compared with *Ch. flavus* were translation, ribosomal structure and biogenesis, amino acid transport and metabolism, and cell wall/membrane biogenesis. The COG categories least increased compared with *Ch. flavus* were transcription, signal transduction mechanisms, and cell motility. Gene order (synteny) was poorly conserved between *Xiphinematobacter* and the five other Verrucomicrobial species with finished (contiguous) genomes (supplementary fig. S5, Supplementary Material online). Further analysis of conserved synteny revealed only 27 blocks with conserved gene order (fig. 3*B*) between free-living outgroups *Ch. flavus*, *O. terrae*, and *P. parvula*. From these blocks, comprising 131 genes, 7/27 synteny blocks were absent in *Xiphinematobacter* and 13/27 blocks, comprising 42 genes, were completely conserved (fig. 3*B*). These most highly conserved synteny blocks consisted of a wide range of well-characterized genes clustered largely by function (e.g., ribosomal structural proteins grouped in block 3, replication genes *gyrB*, *gyrA* in block 5, energy production genes in block 7 *atpD*, *atpG*, *atpA*, etc. [supplementary table S3, Supplementary Material online]), suggestive of conserved operons.

### Pathway and Functional Enrichment (COG and GO)

**Fig. 4.—:**
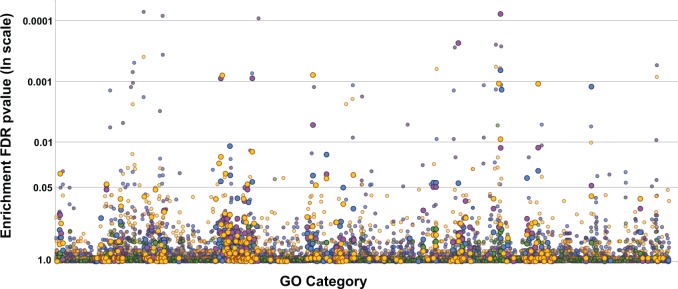
GO category enrichment false discovery rate values (*P* values) showing several clusters of enriched categories for three Verrucomicrobia with reduced genomes, *Xiphinematobacter* (amber circles)*, A. muciniphilia* (purple circles)*, *and *M. infernorum* (blue circles). GO categories (*x*-axis) are sorted by category number and larger circles represent biosynthetic GO categories. By comparison *Wolbachia* wOo (green circles) show lower *P* values and few shared clusters with Verrucomicrobia.

**Fig. 5.—:**
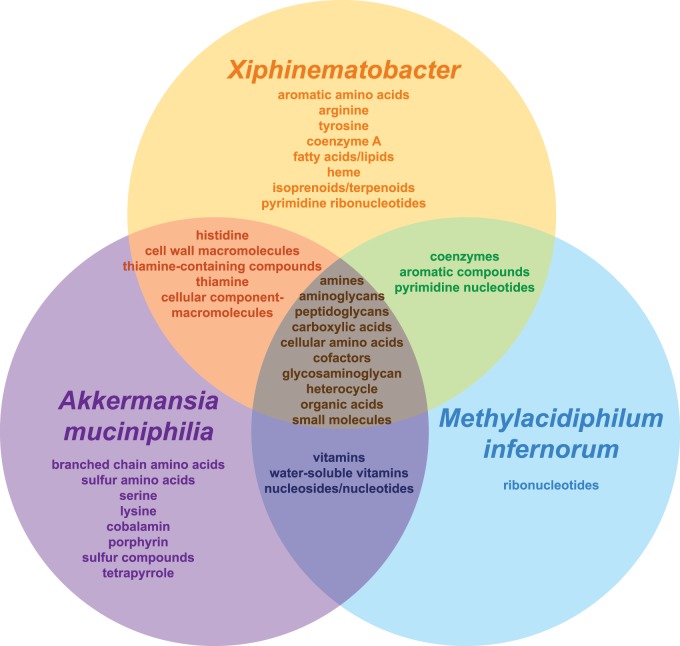
Biosynthetic processes enriched in *Xiphinematobacter, A. muciniphilia*, and *M. infernorum*, compared with a gene set shared between free-living outgroup species *Ch. flavus*, *O. terrae*, and *Pedosphaera parvula*. Similar figures for metabolic/catabolic processes and other processes are in supplementary figure S6, Supplementary Material online.

GO category enrichment, here meaning statistical overrepresentation of a GO term in one genome (e.g., *Xiphinematobacter*) compared with outgroups computed in GoMiner, differed among species (fig. 4; detailed results in supplementary table S7, Supplementary Material online). Although overall amino acid and cofactor biosynthesis was enriched for all three Verrucomicrobia in this study, specific groups of amino acid and cofactor biosynthesis pathways were enriched differently, as shown in figure 5. In particular, *Xiphinematobacter* alone showed enrichment for arginine biosynthesis genes, as well as aromatic amino acid biosynthesis (phenylalanine, tryptophan, and tyrosine). *Akkermansia muciniphilia* alone showed enrichment for serine and lysine biosynthesis, and general sulfur and branched chain amino acid biosynthesis. *Xiphinematobacter* and *A. muciniphilia* were also both enriched for histidine biosynthesis. *Xiphinematobacter* alone was enriched for coenzyme A, fatty acids, lipids, and isoprenoids/terpenoids biosynthesis, and also heme biosynthesis. *Xiphinematobacter* shared enrichment for thiamine (vitamin B1) biosynthesis with *A. muciniphilia* and also shared enrichment for general coenzyme biosynthesis with *M. infernorum*. *Akkermansia muciniphilia* alone was enriched for biosynthesis of cobalamin (vitamin B12) and shared enrichment for general water-soluble vitamin biosynthesis with *M. infernorum*.

**Fig. 6.—:**
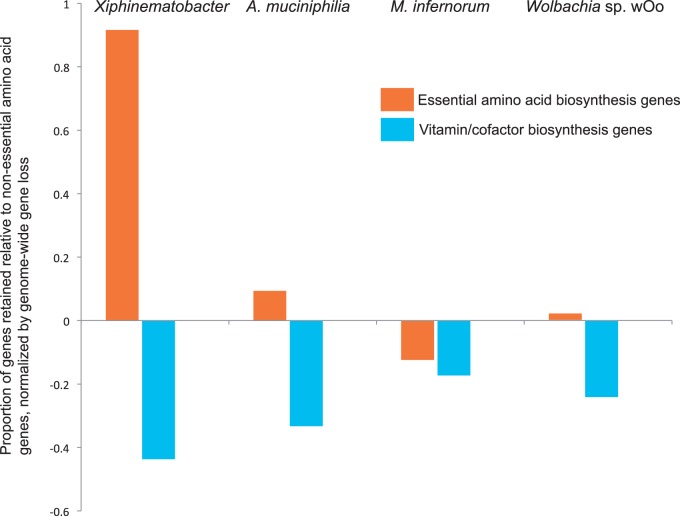
Relative levels of retention of essential amino acid biosynthesis and vitamin/cofactor/coenzyme biosynthesis genes in *Xiphinematobacter, A. muciniphilia, M. infernorum*, and *Wolbachia* wOo, relative to genome-wide gene loss, compared with nonessential amino acid biosynthesis genes.

**Fig. 7.—:**
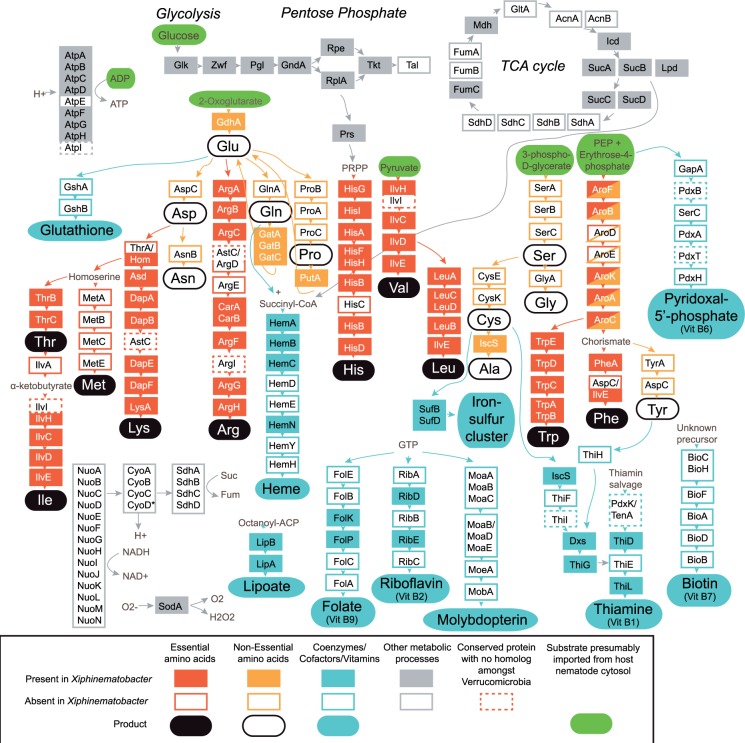
Reconstruction of pathways for biosynthesis of amino acids and vitamins/cofactors, and several other processes in *Xiphinematobacter*, depicting absence of enzymes for de novo synthesis of many amino acids predicted to be nonessential for the host and several vitamins/cofactors, most of which would presumably be imported from the host cytosol (e.g., Asp, Asn, Gln, Pro, Ser, Gly, Cys, Glutathione, Molybdopterin, Biotin, Pyridoxal-5′-phosphate). In contrast, most steps in most amino acid biosynthesis pathways predicted to be essential for the host are present. Glycolysis and pentose phosphate appear to be present, whereas many steps in the TCA cycle are absent.

Because *Xiphinematobacter* is abundant in the gut of the nematode, we performed a comparison to look at potential nutrient supplementation capacity of the symbiont. This comparison looked at predicted essential amino acids and vitamins/cofactors thought to be required in the diet versus predicted nonessential amino acids thought to be readily synthesized de novo by the nematode, based on studies from related nematodes (see Chitwood 1998 and references therein). We found that *Xiphinematobacter* had a larger relative number of essential amino acid genes retained compared with nonessential amino acid biosynthesis genes relative to the other target species (*A. muciniphilia*, *M. infernorum*, and *Wolbachia* wOo) (see fig. 6), when the number of genes in each category was normalized for the genome size reduction in a species relative to the outgroups. The result in figure 6 represents 10 essential amino acid biosynthesis pathways comprising about 130 genes, 11 nonessential amino acid pathways comprising about 50 genes, and 8 vitamins/cofactors or coenzymes pathways comprising 56 genes (details in supplementary table S7, Supplementary Material online). A simplified reconstruction of the retained pathways for biosynthesis of amino acids and vitamins/cofactors in *Xiphinematobacter* shows the retention of most steps in essential amino acid biosynthesis, with the exception of methionine (fig. 7). Conversely, most steps for de novo synthesis of nonessential amino acids and vitamins/cofactors are missing, with the exception of iron–sulfur cluster and lipoate (fig. 7). Very few amino acid or vitamin/cofactor transport genes were present in *Xiphinematobacter*. These included the branched-chain amino acid import genes *livF* and *livH* (but not *livG*, *livM*, *livJ*, and *livK*), the serine/alanine uptake gene *cycA*, and the biotin carboxyl carrier protein *accB*.

A few nonbiosynthetic processes were also enriched in *Xiphinematobacter*; for example, it alone was enriched for terms related to glycolysis/ATP synthesis-coupled proton transport. It was also uniquely enriched for small GTPase-mediated signal transduction, nucleoside/nucleotide interconversion, ribonucleoprotein complex assembly, protein folding/maturation/processing/transport, regulation of cell shape, and defense response to bacterium. *Xiphinematobacter* also shared with *A. muciniphilia* enrichment for terms related to repair (DNA repair, response to DNA damage stimulus) and translation (ribosome biogenesis, translational initiation, tRNA processing). *Xiphinematobacter* shared with *M. infernorum* enrichment for amino acid activation, translational elongation, and tRNA aminoacylation (see full enrichment results in supplementary table S7, Supplementary Material online).

### Incomplete Pathways and Lost or Pseudogenized Genes

The free-living outgroups shared 296 genes that were absent in *Xiphinematobacter*. Among these genes missing in *Xiphinematobacter*, the most common function is regulation (see detailed list in supplementary table S7, Supplementary Material online). For example, 30 of 51 significantly enriched GO terms in the outgroups describe regulation or negative regulation of general biological, metabolic, biosynthetic processes, gene expression and transcription, nitrogen compound metabolism, RNA metabolism, transcription, and nucleotide metabolism. *Xiphinematobacter* also showed significant enrichment for loss of signaling, signal transmission, signal transduction, molybdopterin cofactor biosynthesis and metabolism, prosthetic group metabolism, pteridine-containing compound metabolism and biosynthesis, peptidyl-histidine phosphorylation/modification, peptidyl-amino acid modification, RNA biosynthesis, transcription, and locomotion/taxis/chemotaxis. It also appears that almost all genes involved in mismatch repair, locomotion/chemotaxis, and glutamate and molybdopterin biosynthesis have been lost. Compared with *Ch. flavus*, *Xiphinematobacter* lacks all six genes with predicted cellulase activity. There were four predicted pseudogenes in *Xiphinematobacter*. Among these were thiazole synthase (involved in thiamine synthesis), spore protein SP21 (stress response), and UDP-glucose 4-epimerase (involved in galactose metabolism).

## Discussion

We examined the endosymbiont *Xiphinematobacter* in the plant-parasitic nematode *X. americanum*, showing with FISH microscopy that these bacteria reside in the gut, ovaries, eggs, and embryo gut primordia. We present its complete, annotated genome—the first reported for an exclusively intracellular nematode endosymbiont outside the Genus *Wolbachia*. Comparative genomic analyses show extreme genome reduction and gene set enrichment for several biological functions consistent with this endosymbiont playing a role in nutrition of the host. Most notably, we observed enrichment for animal-essential amino acid biosynthesis, which is consistent with its location in the gut and its host nematode’s likely primary diet of phloem, which is generally depleted in amino acids. We explore how this pattern is widespread among unrelated obligate mutualists in sap-feeding insects, and how this endosymbiosis differs from the mutualisms of *Wolbachia* in filarial nematodes and *A. muciniphilia* in the human colon.

### Key Features Point to Nutritional Mutualism

Our data shed new light on the potential role of *Xiphinematobacter* in its host nematode. Our FISH results at high-stringency confirm features shown previously at lower-stringency (Vandekerckhove et al. 2002) and provide additional clarity on the location, size, and shape of *Xiphinematobacter* cells in adults and developing embryos. Based on previous transmission electron microscopic (TEM) evidence of intracellularity in the ovary and oocytes (Coomans and Claeys 1998; Coomans et al. 2000; Vandekerckhove et al. 2000) several authors suggested *Xiphinematobacter*’s primary role may be to cause thelytokous parthenogenesis in the nematode (Coomans and Claeys 1998; Coomans and Willems 1998; Luc et al. 1998). However, if this were the case, *Xiphinematobacter* could be classified as a reproductive manipulator like most strains of the very distantly related endosymbiont, *Wolbachia*. Our data and previous studies show that like *Wolbachia*, *Xiphinematobacter* has a well-established mechanism for vertical transmission (Vandekerckhove et al. 2002; Werren et al. 2008; Zug and Hammerstein 2014), but unlike reproductive-manipulator strains of *Wolbachia*, it is more localized to specific tissues in its host, with 100% prevalence in all individuals at all stages and at high abundance in the host (Vandekerckhove et al. 2000, 2002). For example, our FISH results confirmed that the symbiont was confined to the ovaries, eggs, and gut wall cells, where the bacteria were actively dividing and abundant. These features are more typical of specialized mutualists, including the mutualist *Wolbachia* strains in nematodes (wBm, wOo, etc.) and insects (wCle) (Foster et al. 2005; Hosokawa et al. 2010; Lefoulon et al. 2012), where the symbiont is required for host survival. Furthermore, if the primary role of *Xiphinematobacter* were to induce parthenogenesis, we must ask why many other species of *Xiphinema* such as *X. index* and *X. pachtaicum*, which also reproduce by parthenogenesis, have not been shown to host the endosymbiont *Xiphinematobacter* (Coomans and Claeys 1998; Coomans et al. 2000). Another topic of interest in our FISH data is the difference in cell shape of *Xiphinematobacter* in the gut versus the ovaries. Our single-nematode sequence results do not support two populations or genotypes, but instead suggest that the single genotype can have two distinct morphologies. It is possible that determination of symbiont shape is under host control, as is seen in other intracellular endosymbionts (Kondorosi et al. 2013), and that differences in cell shape correspond to different functions in these host tissues, but these questions would need to be investigated with further experiments.

Genomic analyses offer more insight into the role of *Xiphinematobacter* in its host nematode, *X. americanum*. Its genome size (0.916 Mb) and number of protein-coding genes (817) are in the range typical for mutualists. For example, genomes of obligate mutualist *Wolbachia* strains from filarial nematodes (including wOo) range from 0.958 to 1.1 Mb, with 647–805 predicted proteins, whereas reproductive parasite *Wolbachia* strains range from 1.3 to 1.6 Mb with 1,010–1,270 proteins (Wu et al. 2004; Foster et al. 2005; Klasson et al. 2008; Comandatore et al. 2013; Duplouy et al. 2013). We also note that *Xiphinematobacter*’s genome is smaller than that from the obligate mutualist *Wolbachia* wCle in the bedbug *Cimex lectularius* (1.25 Mb, with 1,256 genes) (Nikoh et al. 2014). Furthermore, *Xiphinematobacter* falls within the range of most obligate mutualists (genome size < 1.0 Mb) rather than that of facultative symbionts (>1.25 Mb) compared with an array of intracellular symbionts from animals (Moran and Bennett 2014). Although several bacteria with genomes less than 1 Mb are pathogens, notably *Mycoplasma*/*Phytoplasma* at 0.6 Mb, the smallest known genomes, ranging from 0.11 to 0.54 Mb, belong to mutualists, with the majority of species less than 1.0 Mb being endosymbiotic mutualists (NCBI GenBank; April 2015). Furthermore, this extreme genome reduction in mutualists appears to have arisen numerous times, occurring in at least 22 genera from numerous phyla. This pattern could arise from extreme population bottleneck during long-term coevolution with strict vertical transmission, combined with a ratchet effect decreasing the gene set to only those few functions/products or processes that cannot be supplied by the host. Our comparison of genomic features among Verrucomicrobia also fit this pattern, showing much larger genomes and more proteins in species that are host-associated but not obligate mutualists, such as *A. muciniphilia* (in human colon), *Diplosphaera colitermitum* (in termite gut), and *Verrucomicrobiae bacterium DG* (dinoflagellate-associated).

Massive gene loss due to drift is universal in obligate endosymbionts, and can highlight processes that are not necessary in the host environment (e.g., motility). For example, our results suggest that *Xiphinematobacter* has lost many genes for locomotion and taxis/chemotaxis—reflecting an exclusively endosymbiotic lifestyle. Gene loss can also rule-out potential symbiont roles. For example, in *Xiphinematobacter* some functions appear to be completely lost, for example, molybdopterin cofactor and glutamate amino acid biosyntheses, and cellulose degradation. Gene loss can show other changes as well. For example, *Xiphinematobacter* has lost many regulation functions, including negative regulation for biosynthesis of amino acids and vitamins. This is typical for mutualists whose primary role is to synthesize these essential nutrients, and evidence suggests that the host typically takes over the regulation and transport of these products (Dale and Moran 2006; Bennett and Moran 2013).

### Comparative Genomics Suggest Symbiont Role Pathways

Gene set comparisons showed enrichment for several biological processes that illuminate the possible role of *Xiphinematobacter*. Although direct experimental tests of the bacteria’s role are not possible at this time, due to the difficulty in growing *X. americanum* under laboratory conditions, our comparative approach was similar to that used elsewhere (Touchon et al. 2009; Frese et al. 2011; Kjeldsen et al. 2012; Santos-Garcia, Latorre et al. 2014). The basis of our approach was that although theory predicts random mutations cause pseudogenization and subsequent gene loss during bottlenecks, purifying selection will result in enrichment for core housekeeping functions (Herbeck et al. 2003; Touchon et al. 2009). More importantly, within the context of core function enrichment, purifying selection should also enrich for other essential genes and pathways specific to the symbiont’s role in proportion to their influence on fitness (McCutcheon et al. 2009; Williams and Wernegreen 2012; Brown et al. 2014). Our COG comparison with *Ch. flavus*, the closest relative of *Xiphinematobacter* with an available genome, largely fit the first expectation, with the extremely reduced genome of *Xiphinematobacter* having a much lower proportion of poorly annotated genes than *Ch. flavus*—we presume that the genes having no hits to current databases do not serve universally conserved core functions. Furthermore, broad COG categories that had the most and least proportional increase in *Xiphinematobacter* compared with *Ch. flavus* generally represent functions that are most and least universal in bacteria, respectively (e.g., translation, ribosomal structure and biogenesis, cell wall/membrane biogenesis increased most, whereas signal transduction mechanisms, and cell motility increased least). Conversely, our COG comparison suggested that genes involved in transcription were not as well retained as genes involved in amino acid transport and metabolism. This result may reflect the relaxed need for transcription products/processes in the rich environment of the host cell, and the increased need to maintain amino acid transport and metabolism above other core functions, pointing to a possible role in the symbiont. This pattern has been seen in other mutualists (Hansen and Moran 2014; Santos-Garcia, Rollat-Farnier et al. 2014).

Although COG categories show only broad functions, our GO function tests and nutritional pathway reconstruction, provided more specific evidence that *Xiphinematobacter* may function in nutritional supplementation. The basis of these tests was a comparison of distantly related species whose genomes appear to have been reduced under different conditions: Endosymbiosis (*Xiphinematobacter*), extracellular host-association (*A. muciniphilia*), and acidophilic/thermophilic conditions (*M. infernorum*). The tests partially controlled for differences in ancestral metabolism by comparing changed genes only against genes shared among distantly related free-living Verrucomicrobia. The most important result was the enrichment of biosynthesis pathway genes for the amino acid arginine and aromatic amino acids (phenylalanine, tryptophan, and tyrosine) in *Xiphinematobacter* and the increased retention of amino acid biosynthesis genes considered essential in animals, including nematodes, compared with amino acid biosynthesis genes considered nonessential (i.e., that can generally be synthesized de novo). These biosynthetic pathways were not enriched in *A. muciniphilia* or *M. infernorum*, nor in the mutualist *Wolbachia* strain wOo from nematodes.

Free-living bacteria usually synthesize all the common amino acids, whereas intracellular pathogens usually lose the ability to synthesize all amino acids (both essential and nonessential for their hosts) over time (Payne and Loomis 2006). This is due to the high transcriptional and translational cost of these biosynthetic pathways, and Muller’s ratchet under relaxed need for the products, which can be supplied by the host cell. In contrast, obligate mutualists that supply essential amino acids to their animal hosts (e.g., *Buchnera*, *Ishikawaella*, *Sulcia*, *Hodgkinia*, *Carsonella*, *Portiera*, *Evansia*, *Nasuia*, *Zinderia*, etc.) tend to retain these pathways even under conditions of massive genome reduction (Shigenobu et al. 2000; McCutcheon and Moran 2007; Brownlie et al. 2009; Douglas 2009; MacDonald et al. 2011; Nikoh et al. 2011; Santos-Garcia et al. 2012, Santos-Garcia, Latorre et al. 2014, Santos-Garcia, Rollat-Farnier et al. 2014). It is thought that the hosts maintain these symbionts for their ability to supply amino acids or vitamins/cofactors that the hosts cannot de novo synthesize or obtain from their specialized plant sap diets (Sandström and Pettersson 1994). Although we do not explicitly know the metabolic capacity of *X. americanum*, we know that related plant-parasitic nematodes are unable to de novo synthesize the ten essential amino acids and other essential cofactors described for insects and mammals (Barrett 1991; Chitwood 1998). Unlike bacteria-feeding nematodes, which would have ample supply of these nutrients, *X. americanum* has a specialized piercing and sucking feeding structure, the odontostyle that provides a limited liquid diet. Although there are few detailed records of feeding in the *X. americanum* species complex, several observations suggest the diet involves phloem (Cohn 1970; Lamberti and Ciancio 1993). For example, this group feeds exclusively along roots, with the odontostyle often penetrating the stele (or vascular bundle) where the nematode remains in one spot for many hours to days “drinking” using 90 per minute contractions of the esophageal bulb (Cohn 1970), without eliciting a visible host response. This feeding method differs from that of other nematodes related to *Xiphinema* spp. including *Longidorus* spp. and more distantly related Tylenchids, many of which involve modifications of root cortex cells and a notable host response. Access to the vascular bundle (xylem and phloem sieve elements) may be related to the success of *X. americanum* in transmitting nepoviruses, which are abundant in phloem (Kommineni et al. 1998) and presumably move through these tissues.

Interestingly, we found several genes for amino acid or vitamin/cofactor transport/import in *Xiphinematobacter* (*livF*, *livH*, *cycA*, and *accB*), but no homologs for others (*livG*, *livM*, *livJ*, and *livK*), or most amino acid export genes (e.g., *yddG*, *lysE*, *rhtB*, *thrE*, *brnF*, and *brnE*) found in free-living bacteria. It is possible that like other nutrient provisioning endosymbionts with similar sized genomes (e.g., *Buchnera*), the host, rather than the symbiont, may regulate the transport of substrates and products for nutrient biosynthesis (Price et al. 2014). To further explore the role of *Xiphinematobacter* in *X. americanum*, future work might test for signatures of selection on genes in these biosynthesis and transport pathways (e.g., measuring Tajima’s *D*, etc.) using comparative genomics with diverged isolates of this endosymbiont. Our genome-wide comparison of the two strains in this sample showed no obvious functional divergence, probably associated with insufficient time for accumulation/evolution of much nonsynonymous change, based on a low genome-wide Ka/Ks. Alternately, this analysis may show a subtle signal of purifying selection (lower Ka/Ks than the genome-wide average) on the essential amino acid biosynthesis pathways (supplementary fig. S3, Supplementary Material online), although this will need to investigate in future with a range of *Xiphinematobacter* species.

Our enrichment analyses also highlight other potential nutritional roles for *Xiphinematobacter*. For example, biosynthesis of coenzyme A, fatty acids, lipids, isoprenoids/terpenoids and heme, all of which are limited in phloem and essential for nematodes, were enriched, thus could be supplemented by this symbiont (McCutcheon et al. 2009; Moran et al. 2009; Lamelas et al. 2011; Comandatore et al. 2013). Another biosynthetic pathway enriched in *Xiphinematobacter* was the essential amino acid histidine, which was also enriched in *A. muciniphilia*. Histidine biosynthesis requires many genes and is quickly lost in rich host environments when pathogens experience reductive evolution. These two species were also enriched for thiamine (vitamin B1) biosynthesis, suggesting potential similarity in dietary supplementation in *Xiphinematobacter* and *A. muciniphilia* from the human colon. Such similarity might be expected, given that both bacteria are stably associated with the digestive tract of host animals that have dietary need for these essential amino acids and vitamins. Given the proposed role of *A. muciniphilia* in preventing obesity (Everard et al. 2013), our findings illuminate additional potential pathways of interest. Enrichment for most other functions differed between these species.

For some enriched pathways, we found one or more genes were missing (see fig. 7), for example, *hisC* in the histidine pathway and *thiE* and *thiH* in the thiamine biosynthesis pathway and several genes in the heme pathway. Without further data, it is not possible to be certain whether these pathways, which otherwise consist of full-length genes, are intact. We suggest two possibilities for the missing genes: 1) Either the missing genes were recently lost, in which case the pathway is no longer intact and we would expect the remaining genes with no other roles will eventually accumulate mutations and be lost, or 2) a diverged homologous gene or a nonhomologous neofuncionalized gene may take the place of the missing gene and keep the pathway intact. For the missing gene *hisC* in *Xiphinematobacter*, the outgroup Verrucomicrobia species have two diverged *hisC* homologs, raising the possibility of a history of functional divergence in this step of the histidine pathway in these bacteria, and pointing to possibility 2, above, that is, *Xiphinematobacter* having intact histidine biosynthesis. The same case is true for, *hemE* in the heme pathway, whereby outgroups appear to have diverged homologs of this gene, whereas other missing genes such as *hemD*, *hemY*, and *hemH* were annotated in most Verrucomicrobia, but not in *Ch. flavus*, *Xiphinematobacter*’s closest relative in our analysis, suggesting that perhaps diverged analogs could exist. In other cases, missing genes in bifurcating or more complex pathways (e.g., thiamine biosynthesis *thiE* and *thiH*) may reflect an as-yet unclear interaction between host and endosymbiont. Conversely, in cases where all or most genes in a pathway are missing (e.g., the TCA cycle), we favor the first explanation, predicting that probably the process is not intact and we predict that remaining genes without other functions will eventually degrade. In future, support for these predictions could be explored by detailed study of signatures of purifying selection (e.g., Ka/Ks) on these remaining genes in broken pathways by comparison with other species of *Xiphinematobacter*.

The enrichment of coenzyme biosynthesis and amino acid activation in *Xiphinematobacter* are consistent with a role in diet supplementation, as many coenzymes, cofactors, and vitamins are known to be limiting in plant-based diets (Sandström and Pettersson 1994); however, the free-living methanotrophic, thermophilic, acidophile (Hou et al. 2008) *M. infernorum* is also enriched for these. Thus, despite many differences in enrichment profiles for *Xiphinematobacter* and *M. infernorum*, these similarities may be of interest. For example, it has been suggested that *M. infernorum* may have numerous past horizontal gene transfers from the proteobacteria facilitating its lifestyle (Hou et al. 2008), but another possibility is that it descended from ancestors that were symbionts. Indeed, loss of vitamin biosynthesis appears to be universal in eukaryotes and supplementation by microbes is widespread (Helliwell et al. 2013).

### Evidence for Functional Convergence with Endosymbionts of Sap-Feeding Insects

Our comparative genomic results suggest that *Akkermansia*, *Methylacidiphilum, Wolbachia* and *Xiphinematobacter* have different functional profiles, with functional enrichment in *Xiphinematobacter* resembling that found in endosymbionts from sap-feeding insects (Bennett and Moran 2013; Hansen and Moran 2014). The differences between *Xiphinematobacter* and *A. muciniphilia* suggest several potential functions for *A. muciniphilia* that could be explored, particularly, biosynthesis of amino acids serine and lysine and biosynthesis of cobalamin (vitamin B12). It was not surprising to find *M. infernorum* alone was enriched for terms related to inorganic ion transport and energy production, given its free-living status and habitat in geothermal fields. For several reasons, it is also not surprising that the profile of *Xiphinematobacter* differed from that of *Wolbachia* in filarial nematodes. Obviously their food sources are quite different. Additionally, *Wolbachia* has descended from alpha-proteobacteria and appears to have had a very long ancestry as a strictly intracellular symbiont (Weinert et al. 2009; Rodríguez-Ezpeleta and Embley 2012; Nikoh et al. 2014), whereas *Xiphinematobacter* derives from Verrucomicrobial lineages that appear to be more recently adapted from free-living ancestors (Vandekerckhove et al. 2000). In future, more detailed comparisons of gene set enrichment among strains of *Wolbachia* could shed light on the evolution of functional roles in this group, but given the very large evolutionary distance and host differences between *Xiphinematobacter* and *Wolbachia*, we predict limited overlap. Of perhaps greater interest is a future analysis of *Wolbachia* species from plant-parasitic nematodes (Haegeman et al. 2009), for which we might expect some gene set convergence with *Xiphinematobacter*, if they function as nutritional mutualists. However, at present genomes are not available from this group.

Our study adds to the growing list of bacterial phyla that appear to have independently converged on nutritional supplementation in their hosts (McCutcheon and Moran 2010; Toft and Andersson 2010; Nikoh et al. 2014). Although nutritional supplementation can take on various forms, here the functional profile of *Xiphinematobacter* most closely matched that from bacterial mutualists in phloem-feeding insects (e.g., *Buchnera*) (Hansen and Moran 2014). We suggest that the common selective force on these endosymbionts relates to the need to supplement limiting essential amino acids in the plant-fluid restricted diet. Of course, this restricted diet is a result of the specialized mouthparts for piercing and sucking (odontostyle in *Xiphinema* nematodes or stylet in Hemipteran insects). In Hemipterans, the sap-feeding through stylet arose perhaps 270 Ma (see Bennett and Moran 2013 and references therein), along with a dependence on obligate bacterial mutualists to supplement nutrients in the sap-diet. In the Longidorid nematodes, the odontostyle and the occurrence of the endosymbiont *Xiphinematobacter* arose perhaps 50–100 Ma (Vandekerckhove et al. 2000). This more recent history of endosymbiosis could explain some genomic features observed in *Xiphinematobacter*, such as intermediate %GC reduction, intermediate tRNA gene number compared with obligate mutualists from Hemiptera. These features could also result from weaker selection due to less strict dependence on phloem in the diet of *X. americanum*; however, details of the feeding mode and diet of *X. americanum* need to be better characterized. Taxonomic challenges across the broad *X. americanum* species-complex (Lamberti and Ciancio 1993; Zasada et al. 2014) and incomplete study of the occurrence of *Xiphinematobacter* in these hosts make it difficult correlate feeding mode and symbiont for this group. The early appearance of the endosymbiont in gut primordia of first-stage juveniles before hatching also raises the question of nutrient supplementation variance during the nematode lifespan.

### Nutritional Mutualists in Nematodes: Widespread or Rare?

It is not clear how widespread nutritional mutualism is among nematodes, but our study of *Xiphinematobacter* tends to support previous results showing that among known nematode-bacterial symbioses, diet specialization may be a predictor. For example, several extracellular ectosymbionts, gut lumen, or other organ mutualists have been described in nematodes (e.g., *Photorhabdus*, *Xenorhabdus*, and various microbiome inhabitants) (Burnell and Stock 2000; Bayer et al. 2009; Baquiran et al. 2013; Cheng et al. 2013), all of which are associated with some sort of nutrient provisioning. *Photorhabdus* and *Xenorhabdus*, bacterial symbionts of entomopathogenic nematodes, have quite specialized diet and associations with their hosts, but unlike *Xiphinematobacter*, have obligate stages outside the nematode cells and retain much larger genomes (Chaston et al. 2011). Several exclusively intracellular endosymbionts are known, such as *Wolbachia* strains in parasitic filarial nematodes (Foster et al. 2005; Lefoulon et al. 2012; Landmann et al. 2014) and the plant-parasitic nematode *Radopholus similis* (Haegeman et al. 2009), and *Wolbachia* genetic remnants suggestive of a possible past endosymbiosis in parasitic strongyloidean nematodes (Koutsovoulos et al. 2014). Others include *Cardinium* strains in the plant-parasitic nematodes *Globodera* and *Heterodera* (Noel and Atibalentja 2006), *Comamonas* sp. in parasitic spiruridean nematodes (Gottlieb et al. 2012), and an undescribed bacteria in the nematode *Noctuidonema* that are ectoparasites of insects (Marti et al. 1995). We speculate that within the *X. americanum* species-complex, members which depend most on phloem might depend more on this bacterial partner, whereas species within or outside of the *X. americanum* group that do not feed on phloem may not need this symbiont at all. Given the often overlooked diversity of nematodes themselves (De Ley 2006), we speculate that there may be many other important nutritional mutualist endosymbionts to be discovered in the Nematoda.

## Conclusions

Using a comparative approach we analyzed the first genome from an exclusively intracellular nematode endosymbiont, other than *Wolbachia*, and showed evidence that the Verrucomicrobial endosymbiont *Xiphinematobacter* likely serves as a nutritional mutualist for its host *X. americanum. *Specific evidence includes location in the gut and gut primordia of the embryo, massive genome reduction that mirrors that found in obligate intracellular mutualists, and enrichment for retention of essential amino acid and vitamin biosynthesis pathways. Explicit demonstration of this role awaits experimental testing with and without the symbiont. Although such manipulative experiments are not feasible at present given the difficulty of growing these nematodes in the laboratory, our results illuminate promising directions for future work and management of the plant-parasitic nematode *X. americanum* species-complex. For example, these findings suggest candidate limiting nutrient pathways that may explain nematode specialization or pathogenicity on various plants, and suggest alternative nonnematicidal targets for control. This study is novel in presenting the first genome from an endosymbiotic Verrucomicrobia, which showed perhaps unexpected similarity to genomes from endosymbionts of strictly sap-feeding Hemiptera—a pattern of convergent strategies in symbiont potentially resulting from convergence in feeding mode. Moreover, our findings highlight how much is still to be learned about the broader diversity, abundance, and functional relationships of nematode bacterial symbionts.

## Supplementary Material

Supplementary figures S1–S6 and tables S1–S7 are available at *Genome Biology and Evolution *online (http://www.gbe.oxfordjournals.org/).

Supplementary Data
