# Supplementary material for: Comparative Genomics of a Plant-Parasitic Nematode Endosymbiont Suggest a Role in Nutritional Symbiosis
Source: Genome Biol Evol. 2015 Sep 10;7(9):2727–46. doi: 10.1093/gbe/evv176 (PMC4607532; doi:10.1093/gbe/evv176)
Supplement: Supplementary Data [file supp_7_9_2727__index.html]

Comparative Genomics of a Plant-Parasitic Nematode Endosymbiont Suggest a Role in Nutritional Symbiosis — Supplementary Data 

# Comparative Genomics of a Plant-Parasitic Nematode Endosymbiont Suggest a Role in Nutritional Symbiosis

## Supplementary Data

files

- Supplementary Data - zip file
