# Supplementary material for: Comparative Genomics of a Plant-Parasitic Nematode Endosymbiont Suggest a Role in Nutritional Symbiosis
Source: Genome Biol Evol. 2015 Sep 10;7(9):2727–46. doi: 10.1093/gbe/evv176 (PMC4607532; doi:10.1093/gbe/evv176)
Supplement: Supplementary Data [file supp_evv176_suppl_data.zip › SupplFigures1to6.pdf]

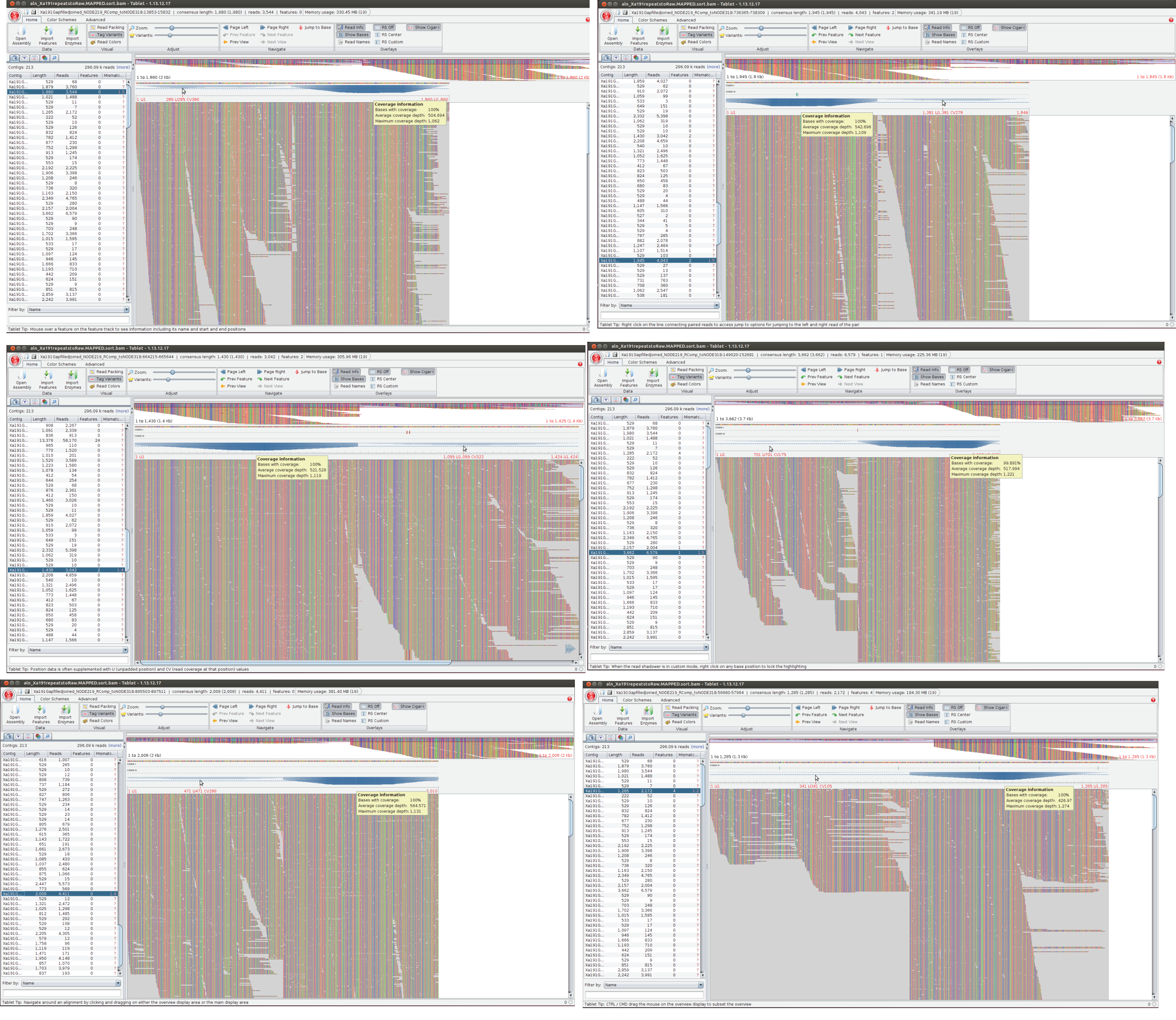
aln_Xa191repeatstoRaw.MAPPED.sort.bam - Tablet - 1.13.12.17
Xa191GapfilledJoined_NODE219_RComp_toNODE318:13853-15832 | consensus length: 1,980 (1,980) | reads: 3,544 | features: 0 | Memory usage: 330.45 MB (19)
Coverage information
Bases with coverage: 100%
Average coverage depth: 504.694
Maximum coverage depth: 1,062
aln_Xa191repeatstoRaw.MAPPED.sort.bam - Tablet - 1.13.12.17
Xa191GapfilledJoined_NODE219_RComp_toNODE318:736365-738309 | consensus length: 1,945 (1,945) | reads: 4,043 | features: 2 | Memory usage: 341.19 MB (19)
Coverage information
Bases with coverage: 100%
Average coverage depth: 542.696
Maximum coverage depth: 1,109
aln_Xa191repeatstoRaw.MAPPED.sort.bam - Tablet - 1.13.12.17
Xa191GapfilledJoined_NODE219_RComp_toNODE318:664215-665644 | consensus length: 1,430 (1,430) | reads: 3,042 | features: 2 | Memory usage: 305.96 MB (19)
Coverage information
Bases with coverage: 100%
Average coverage depth: 521.529
Maximum coverage depth: 1,119
aln_Xa191repeatstoRaw.MAPPED.sort.bam - Tablet - 1.13.12.17
Xa191GapfilledJoined_NODE219_RComp_toNODE318:149020-152681 | consensus length: 3,662 (3,662) | reads: 6,579 | features: 1 | Memory usage: 225.36 MB (19)
Coverage information
Bases with coverage: 99.891%
Average coverage depth: 517.994
Maximum coverage depth: 1,221
aln_Xa191repeatstoRaw.MAPPED.sort.bam - Tablet - 1.13.12.17
Xa191GapfilledJoined_NODE219_RComp_toNODE318:895503-897511 | consensus length: 2,009 (2,009) | reads: 4,411 | features: 0 | Memory usage: 381.40 MB (19)
Coverage information
Bases with coverage: 100%
Average coverage depth: 564.571
Maximum coverage depth: 1,131
aln_Xa191repeatstoRaw.MAPPED.sort.bam - Tablet - 1.13.12.17
Xa191GapfilledJoined_NODE219_RComp_toNODE318:56680-57964 | consensus length: 1,285 (1,285) | reads: 2,172 | features: 4 | Memory usage: 184.30 MB (19)
Coverage information
Bases with coverage: 100%
Average coverage depth: 426.97
Maximum coverage depth: 1,274

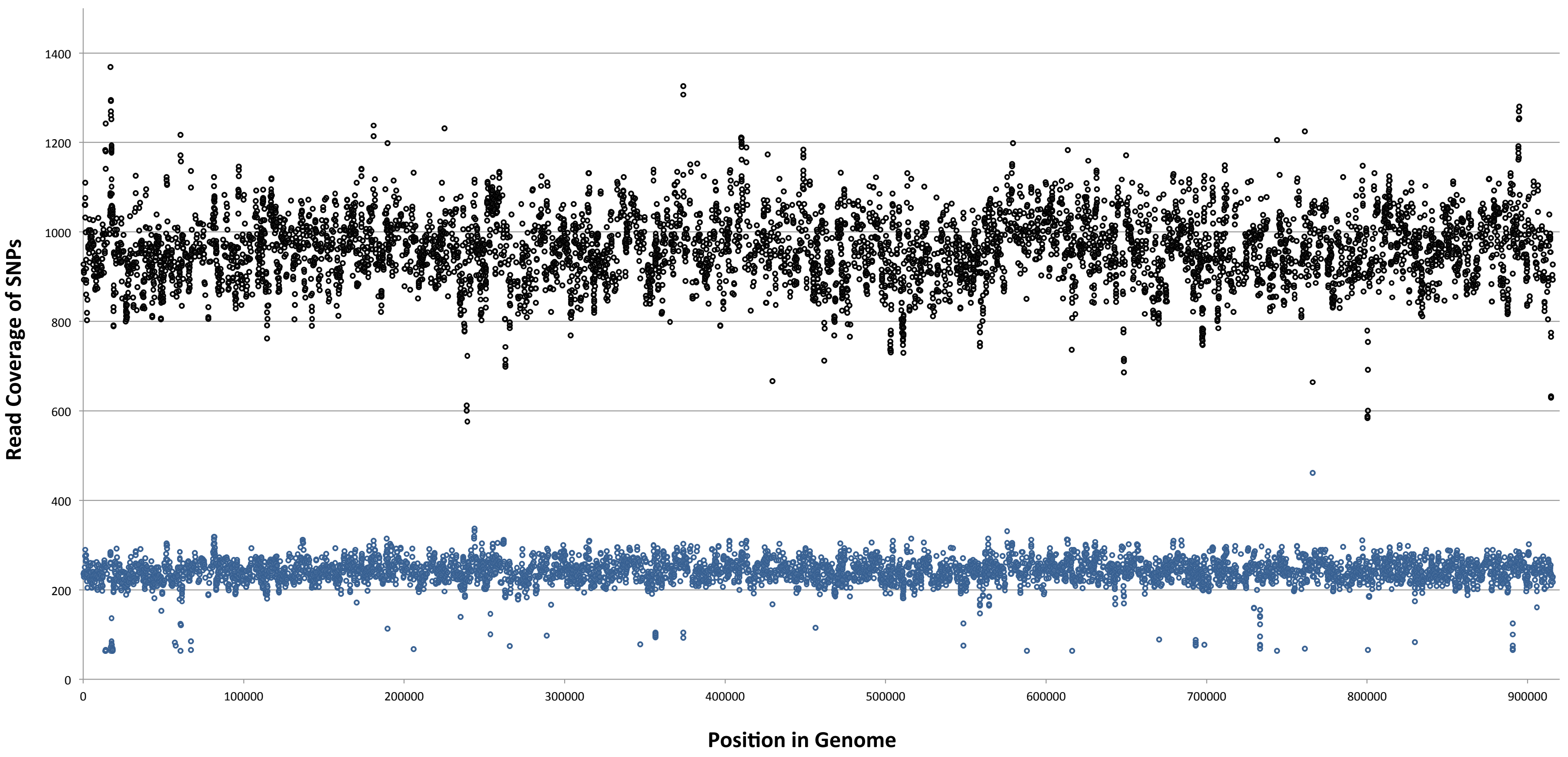

Read Coverage of SNPs
Position in Genome
0
200
400
600
800
1000
1200
1400
0
100000
200000
300000
400000
500000
600000
700000
800000
900000

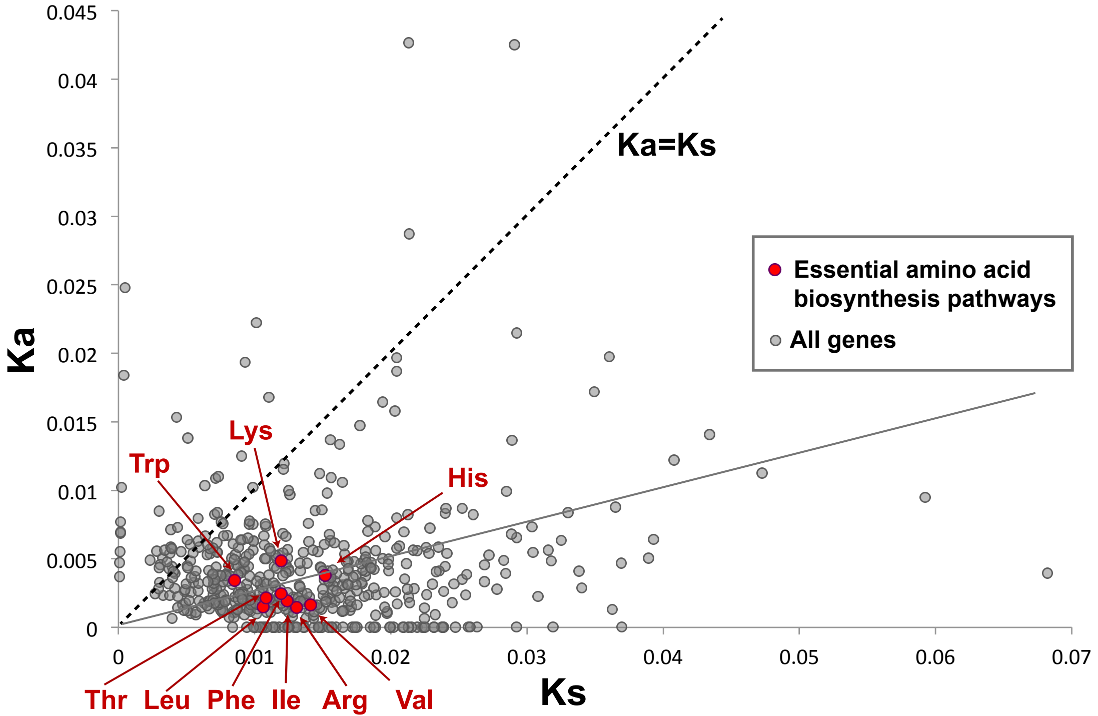

Ka
Ks
0.045
0.04
0.035
0.03
0.025
0.02
0.015
0.01
0.005
0
0
0.01
0.02
0.03
0.04
0.05
0.06
0.07
Ka=Ks
Essential amino acid biosynthesis pathways
All genes
Lys
Trp
His
Thr
Leu
Phe
Ile
Arg
Val

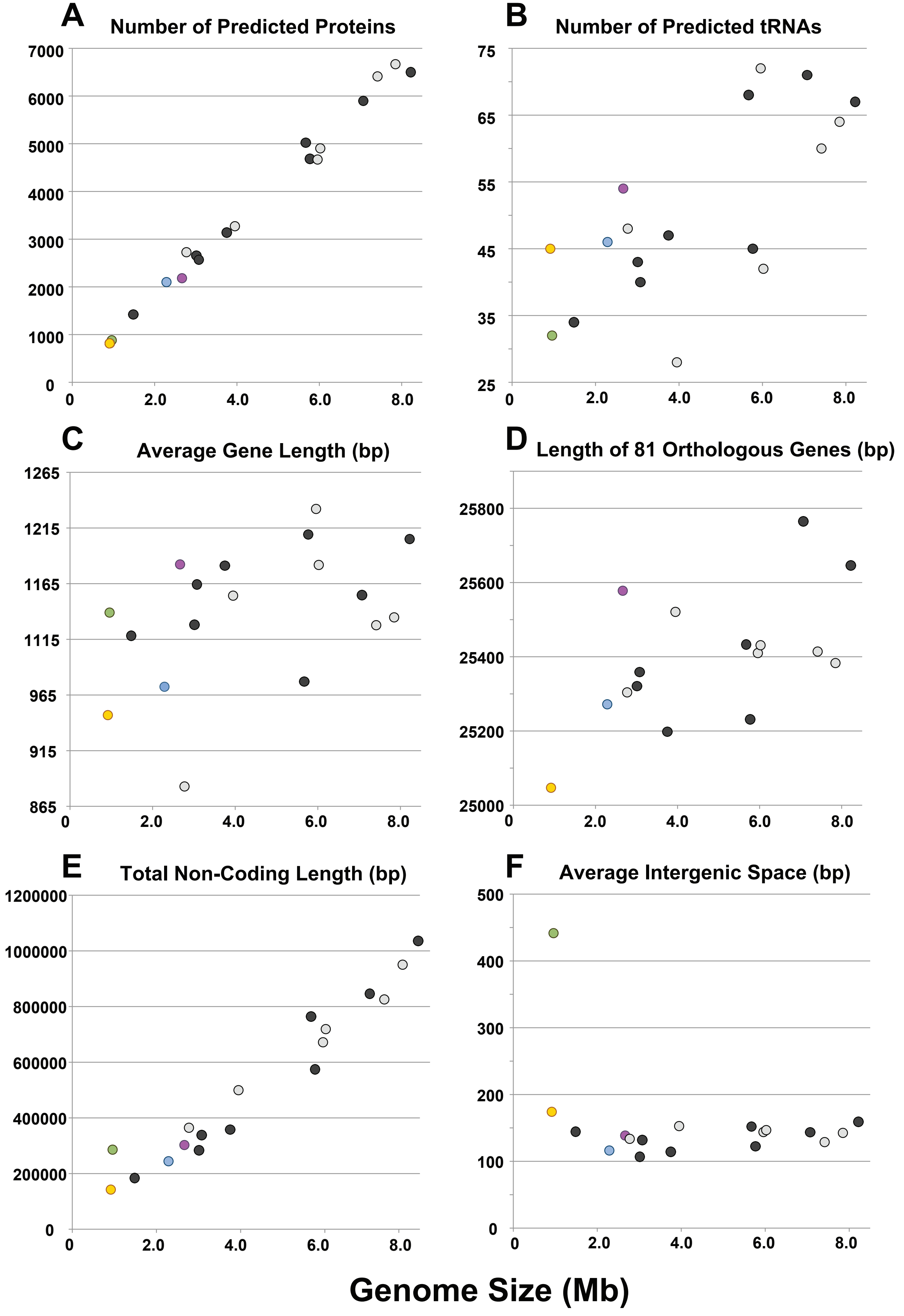
A
Number of Predicted Proteins
0
1000
2000
3000
4000
5000
6000
7000
B
Number of Predicted tRNAs
25
35
45
55
65
75
C
Average Gene Length (bp)
865
915
965
1115
1165
1215
1265
D
Length of 81 Orthologous Genes (bp)
25000
25200
25400
25600
25800
E
Total Non-Coding Length (bp)
0
200000
400000
600000
800000
1000000
1200000
F
Average Intergenic Space (bp)
0
100
200
300
400
500
0
2.0
4.0
6.0
8.0
Genome Size (Mb)

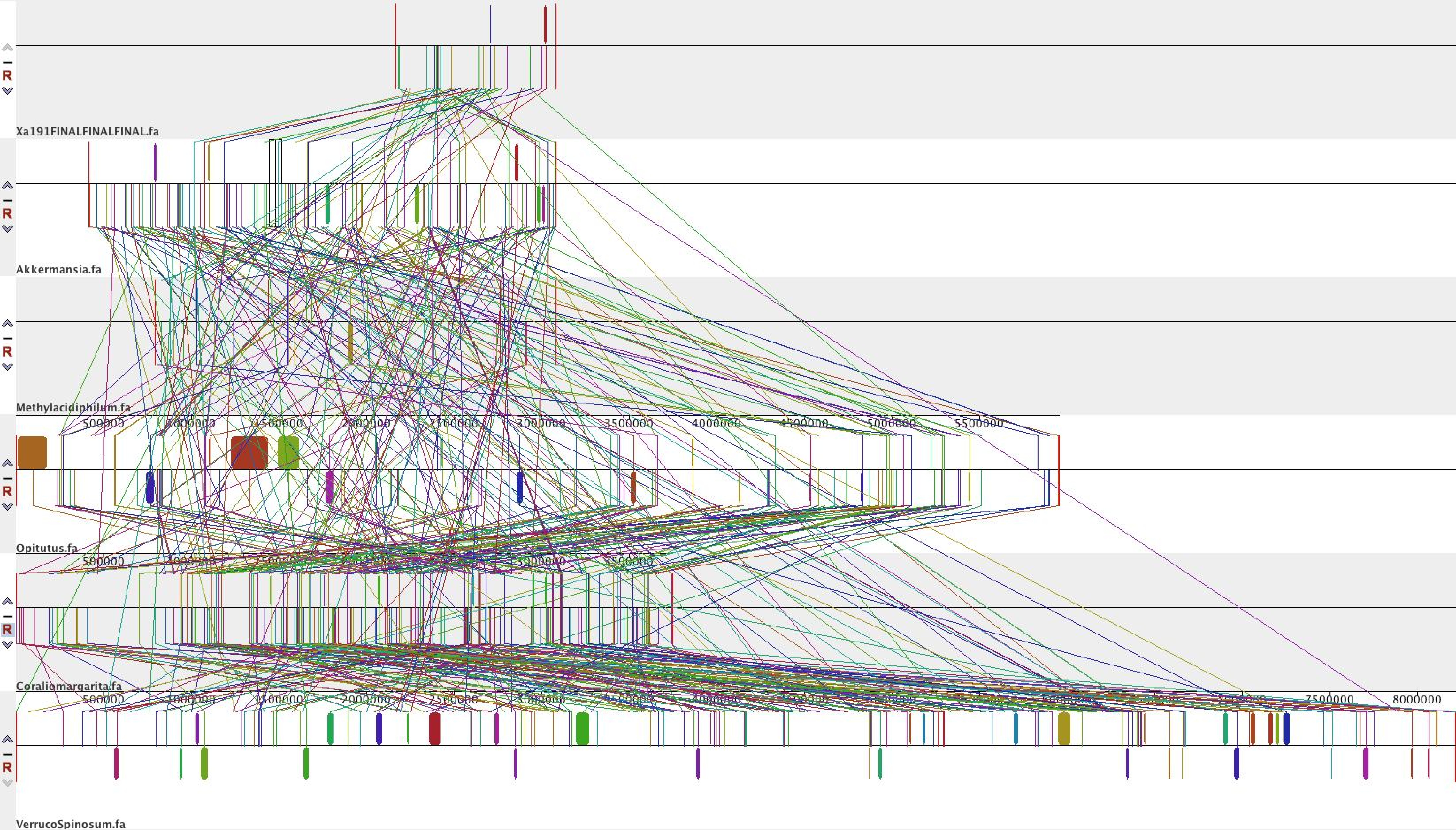
Xa191FINALFINALFINAL.fa
Akkermansia.fa
Methylacidiphilum.fa
500000
1000000
1500000
2000000
2500000
3000000
3500000
4000000
4500000
5000000
5500000
Opitutus.fa
Coraliomargarita.fa
7500000
8000000
VerrucoSpinosum.fa

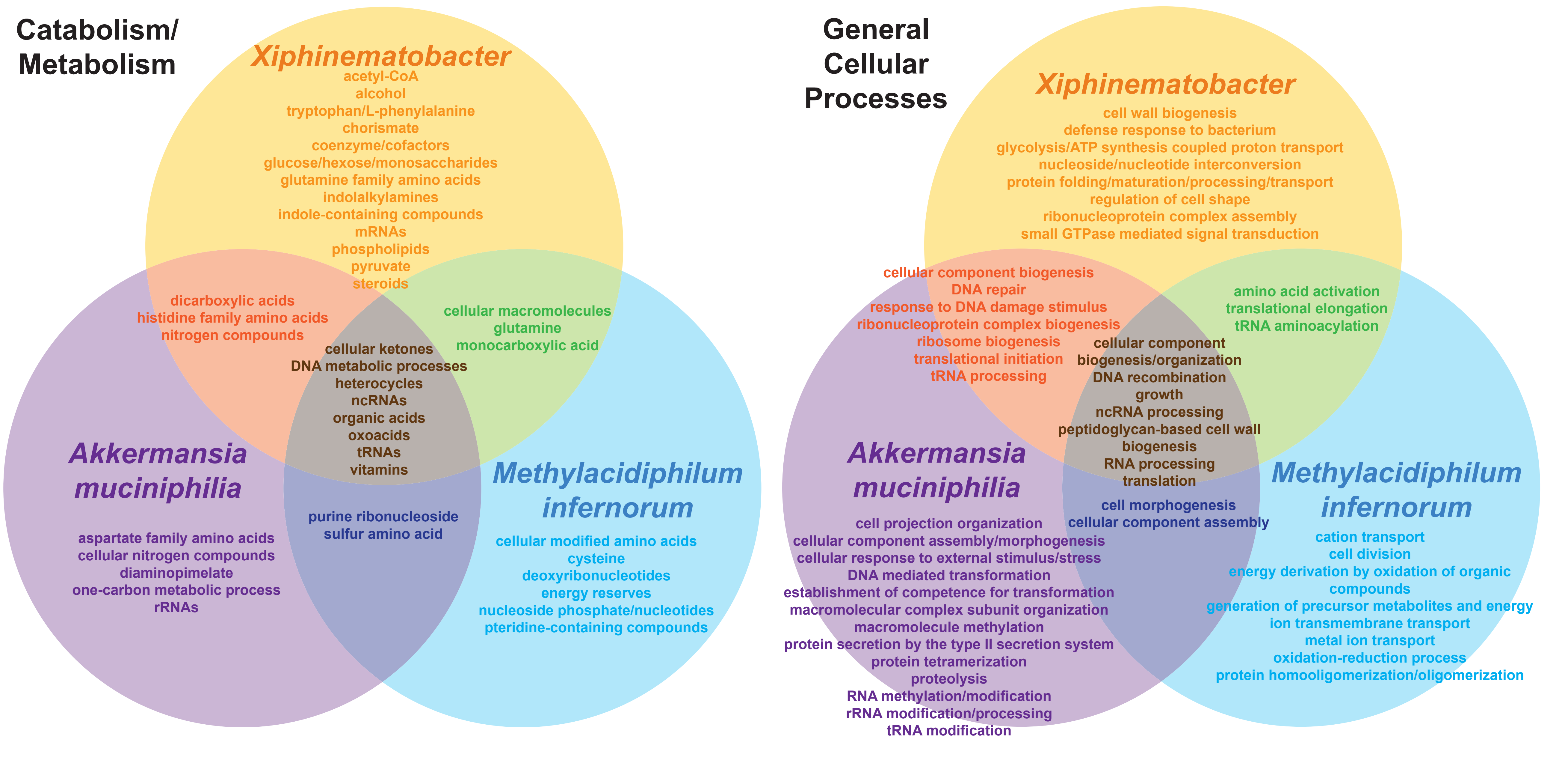

Catabolism/ Metabolism
Xiphinematobacter
acetyl-CoA
alcohol
tryptophan/L-phenylalanine
chorismate
coenzyme/cofactors
glucose/hexose/monosaccharides
glutamine family amino acids
indolalkylamines
indole-containing compounds
mRNAs
phospholipids
pyruvate
steroids
dicarboxylic acids
histidine family amino acids
nitrogen compounds
cellular macromolecules
glutamine
monocarboxylic acid
cellular ketones
DNA metabolic processes
heterocycles
ncRNAs
organic acids
oxoacids
tRNAs
vitamins
Akkermansia muciniphilia
aspartate family amino acids
cellular nitrogen compounds
diaminopimelate
one-carbon metabolic process
rRNAs
purine ribonucleoside
sulfur amino acid
Methylacidiphilum infernorum
cellular modified amino acids
cysteine
deoxyribonucleotides
energy reserves
nucleoside phosphate/nucleotides
pteridine-containing compounds
General Cellular Processes
Xiphinematobacter
cell wall biogenesis
defense response to bacterium
glycolysis/ATP synthesis coupled proton transport
nucleoside/nucleotide interconversion
protein folding/maturation/processing/transport
regulation of cell shape
ribonucleoprotein complex assembly
small GTPase mediated signal transduction
cellular component biogenesis
DNA repair
response to DNA damage stimulus
ribonucleoprotein complex biogenesis
ribosome biogenesis
translational initiation
tRNA processing
amino acid activation
translational elongation
tRNA aminoacylation
cellular component biogenesis/organization
DNA recombination
growth
ncRNA processing
peptidoglycan-based cell wall biogenesis
RNA processing
translation
Akkermansia muciniphilia
cell projection organization
cellular component assembly/morphogenesis
cellular response to external stimulus/stress
DNA mediated transformation
establishment of competence for transformation
macromolecular complex subunit organization
macromolecule methylation
protein secretion by the type II secretion system
protein tetramerization
proteolysis
RNA methylation/modification
rRNA modification/processing
tRNA modification
cell morphogenesis
cellular component assembly
Methylacidiphilum infernorum
cation transport
cell division
energy derivation by oxidation of organic compounds
generation of precursor metabolites and energy
ion transmembrane transport
metal ion transport
oxidation-reduction process
protein homooligomerization/oligomerization
