# Supplementary material for: Comparative Genomics of a Plant-Parasitic Nematode Endosymbiont Suggest a Role in Nutritional Symbiosis
Source: Genome Biol Evol. 2015 Sep 10;7(9):2727–46. doi: 10.1093/gbe/evv176 (PMC4607532; doi:10.1093/gbe/evv176)
Supplement: Supplementary Data [file supp_evv176_suppl_data.zip › SupplTablesCombined.pdf]

Supplementary Table S1. Primers used for PCR-finishing of *Xiphinematobacter* genome and amplifying the variable NAD synthase region in *Xiphinematobacter* isolates from single nematodes.

Primer	Sequence 5' to 3'	Regions Amplified
**ES1F**[1]	GTTCTTCAACGTATCGCAACTTT	Closure of circle (659 bp)
**ES4R**[1]	AAACGTTTGCGCAGAGTGGCCT	
**CJ2F**[2]	CTGAAGTATAGACTATTCAACTG	Junction between contigs (1.6 kb)
**CJ4R**[2]	ACTCTATTATATTCCGTATTCCG	
**NAD1-F**[3]	CCCACGATGGCGGGCTTCATTTA	NAD synthase region (740 bp)[4]
**NAD1-R**[3]	ATCTCTCTGGTAGCCTGTAGCCC	from single nematodes[5]

[1,2] Conditions: 96°C - 2 min, 30 cycles of: 96°C - 30 sec, 55°C – 30 sec, 72°C – 2 min, 72°C - 5min. [3] Conditions: 96°C - 2 min, 30 cycles of: 96°C - 30 sec, 60°C – 30 sec, 72°C – 1 min 45 sec, 72°C - 5min. [4] *Xiphinematobacter* region includes 117 bp of the 5' end of arginine carboxylase, 285 bp of intergenic space, and 338 bp of the 5' end of NAD synthase. [5] Single-nematode DNA preparation was performed following (Zasada et al. 2014), with an additional step to ensure breaking the nemtode cuticle using a glass pipette melted slightly to act as a modified pestle.

Zasada IA, Peetz A, Howe DK, Wilhelm LJ, Cheam D, Denver DR, Smythe AB. 2014. Using mitogenomic and nuclear ribosomal sequence data to investigate the phylogeny of the *Xiphinema americanum* species complex. PLoS One 9:e90035.

Supplementary Table S2. List of Predicted Signal Peptides and Pseudogenes in *Xiphinematobacter*.

Predicted Signal Peptides in *Xiphinematobacter*:

Gene	Description	Evidence
folK	2-amino-4-hydroxy-6-hydroxymethyldihydropteridine pyrophosphokinase	predicted cleavage at residue 47 with probability 0.982
atpE	ATP synthase subunit c	predicted cleavage at residue 29 with probability 0.876
PROKKA_00571	CAAX amino terminal protease self- immunity	predicted cleavage at residue 20 with probability 0.765
pgsA	CDP-diacylglycerol--glycerol-3-phosphate 3-phosphatidyltransferase	predicted cleavage at residue 22 with probability 0.920
mreC	Cell shape-determining protein MreC precursor	predicted cleavage at residue 28 with probability 0.643
coaBC_1	Coenzyme A biosynthesis bifunctional protein CoaBC	predicted cleavage at residue 22 with probability 0.690
ccs1	Cytochrome c biogenesis protein Ccs1	predicted cleavage at residue 34 with probability 0.948
dsbH	Disulfide bond reductase DsbH precursor	predicted cleavage at residue 20 with probability 0.994
PROKKA_00046	hypothetical protein	predicted cleavage at residue 28 with probability 0.968
PROKKA_00158	hypothetical protein	predicted cleavage at residue 23 with probability 0.795
PROKKA_00246	hypothetical protein	predicted cleavage at residue 28 with probability 0.734
PROKKA_00404	hypothetical protein	predicted cleavage at residue 31 with probability 0.746
PROKKA_00445	hypothetical protein	predicted cleavage at residue 38 with probability 0.731
PROKKA_00519	hypothetical protein	predicted cleavage at residue 44 with probability 0.751
PROKKA_00531	hypothetical protein	predicted cleavage at residue 23 with probability 0.509
PROKKA_00609	hypothetical protein	predicted cleavage at residue 30 with probability 0.753
PROKKA_00685	hypothetical protein	predicted cleavage at residue 34 with probability 0.666
PROKKA_00823	hypothetical protein	predicted cleavage at residue 23 with probability 0.744
ftsW	Lipid II flippase FtsW	predicted cleavage at residue 35 with probability 1.000
corC_1	Magnesium and cobalt efflux protein CorC	predicted cleavage at residue 34 with probability 0.979
nlpD	Murein hydrolase activator NlpD precursor	predicted cleavage at residue 29 with probability 0.918
PROKKA_00251	Outer membrane protein (OmpH-like)	predicted cleavage at residue 25 with probability 1.000
pal	Outer membrane protein P6 precursor	predicted cleavage at residue 27 with probability 0.966
pstS	Phosphate-binding protein PstS precursor	predicted cleavage at residue 23 with probability 0.915
cdsA	Phosphatidate cytidylyltransferase	predicted cleavage at residue 28 with probability 0.916
oprB	Porin B precursor	predicted cleavage at residue 38 with probability 0.991
PROKKA_00081	preprotein translocase subunit YajC	predicted cleavage at residue 18 with probability 0.814
cbf2	Putative peptidyl-prolyl cis-trans isomerase Cbf2 precursor	predicted cleavage at residue 23 with probability 0.877
PROKKA_00384	putative periplasmic iron-binding protein precursor	predicted cleavage at residue 22 with probability 0.988
hhoB	Putative serine protease HhoB precursor	predicted cleavage at residue 21 with probability 0.660
PROKKA_00686	SigmaW regulon antibacterial	predicted cleavage at residue 28 with probability 0.712
spoVD_2	Stage V sporulation protein D	predicted cleavage at residue 23 with probability 0.501
tolB	tolB	predicted cleavage at residue 20 with probability 0.992

Possible Pseudogenes in *Xiphinematobacter*:

Possible /pseudo 'von Willebrand factor type A domain protein' at Xa191FinalGapfilledSanger position 261656
Possible /pseudo 'Thiazole synthase' at Xa191FinalGapfilledSanger position 629631
Possible /pseudo 'Spore protein SP21' at Xa191FinalGapfilledSanger position 696891
Possible /pseudo 'UDP-glucose 4-epimerase' at Xa191FinalGapfilledSanger position 856370
