# Supplementary material for: Comparative Genomics of a Plant-Parasitic Nematode Endosymbiont Suggest a Role in Nutritional Symbiosis
Source: Genome Biol Evol. 2015 Sep 10;7(9):2727–46. doi: 10.1093/gbe/evv176 (PMC4607532; doi:10.1093/gbe/evv176)
Supplement: Supplementary Data [file supp_evv176_suppl_data.zip › New Microsoft Office Word Document.docx]

**Supplementary Materials:**

**Supplementary figure S1.** Some examples of read coverage ratio for duplicated polymorphic repeats from initial assembly of *Xiphinematobacter* from screen shots in Tablet Genome Viewer.

**Supplementary table S1.** Primers used for PCR-finishing the *Xiphinematobacter* genome.

**Supplementary table S2.** Predicted signal peptides and pseudogenes in *Xiphinematobacter*.

**Supplementary table S3.** Synteny data used in figure 4B.

**Supplementary table S4.** Single nucleotide polymorphism and indel data for second *Xiphinematobacter* strain.

**Supplementary figure S2.** Plot showing SNP frequency data for common and rare strains in the *Xiphinematobacter* Illumina sequence data.

**Supplementary figure S3.** Plot showing the rates of non-synonymous (Ka) and synonymous (Ks) substitutions for all genes individually (grey dots) and for genes in essential amino acid pathways (red dots) for the two *Xiphinematobacter* strains.

**Supplementary table S5.** List of orthologous genes compared in phylogenomic tree of Verrucomicrobia and outgroups and translated protein sequence alignment for 81 orthologous genes, in multi fasta format.

**Supplementary figure S4.** Various genomic features plotted against genome size in Mbp on x-axis for the species in table 2. Host-associated species, *Xiphinematobacter* (amber circles), *Akkermansia muciniphilia* (purple circles)*,* and *Wolbachia* wOo (green circles), and others (dark circles), or free-living species *Methylacidiphilum infernorum* (blue circles)*,* and others (open circles).

**Supplementary table S6.** Amino acid alignment used for constructing phylogenetic tree shown in figure 2.

**Supplementary figure S5.** Genomic synteny pattern predicted in Mauve for *Xiphinematobacter* and other Verrucomicrobia with finished genomes.

**Supplementary table S7.** Gene ontology category enrichment test results for (1) genes from *Xiphinematobacter*, *Akkermansia muciniphilia*, and *Methylacidiphilum infernorum*, compared with genes shared amongst free-living outgroup Verrucomicrobia; (2) genes from *Wolbachia* wOo, compared with genes from *Wolbachia* wPip; (3) genes shared amongst free-living outgroup Verrucomicrobia, compared with genes from *Xiphinematobacter*. Also includes list of genes in amino acid and cofactor biosynthesis pathways in comparative analysis shown in figure 7.

**Supplementary figure S6.** Enrichment for metabolic/catabolic and other processes for *Xiphinematobacter*, *Akkermansia muciniphilia*, and *Methylacidiphilum infernorum*.
